# Excess phosphoserine-129 α-synuclein induces synaptic vesicle trafficking and declustering defects at a vertebrate synapse

**DOI:** 10.1091/mbc.E23-07-0269

**Published:** 2023-11-22

**Authors:** Jaqulin N. Wallace, Zachary C. Crockford, Cristina Román-Vendrell, Emily B. Brady, Christian Hoffmann, Karina J. Vargas, Mariana Potcoava, M. Elizabeth Wegman, Simon T. Alford, Dragomir Milovanovic, Jennifer R. Morgan

**Affiliations:** a Eugene Bell Center for Regenerative Biology and Tissue Engineering, and; c Whitman Center, Marine Biological Laboratory, Woods Hole, MA 02543; b Laboratory of Molecular Neuroscience, German Center for Neurodegenerative Diseases (DZNE), 10117 Berlin, Germany; d Department of Cell Biology, University of Pittsburgh, PA 15261; e Department of Anatomy and Cell Biology, University of Illinois at Chicago, Chicago, IL 60612; Brandeis University

## Abstract

α-Synuclein is a presynaptic protein that regulates synaptic vesicle (SV) trafficking. In Parkinson’s disease (PD) and dementia with Lewy bodies (DLB), α-synuclein aberrantly accumulates throughout neurons, including at synapses. During neuronal activity, α-synuclein is reversibly phosphorylated at serine 129 (pS129). While pS129 comprises ∼4% of total α-synuclein under physiological conditions, it dramatically increases in PD and DLB brains. The impacts of excess pS129 on synaptic function are currently unknown. We show here that compared with wild-type (WT) α-synuclein, pS129 exhibits increased binding and oligomerization on synaptic membranes and enhanced vesicle “microclustering” in vitro. Moreover, when acutely injected into lamprey reticulospinal axons, excess pS129 α-synuclein robustly localized to synapses and disrupted SV trafficking in an activity-dependent manner, as assessed by ultrastructural analysis. Specifically, pS129 caused a declustering and dispersion of SVs away from the synaptic vicinity, leading to a significant loss of total synaptic membrane. Live imaging further revealed altered SV cycling, as well as microclusters of recently endocytosed SVs moving away from synapses. Thus, excess pS129 caused an activity-dependent inhibition of SV trafficking via altered vesicle clustering/reclustering. This work suggests that accumulation of pS129 at synapses in diseases like PD and DLB could have profound effects on SV dynamics.

## Abstract (executive summary)

Levels of phosphoserine 129 (pS129) α-synuclein increase dramatically in Parkinson’s disease and other synucleinopathies, but the impacts on synapses are unknown.

Here, we demonstrate that excess pS129 α-synuclein alters synaptic vesicle clustering dynamics in vitro and in vivo, resulting in synaptic phenotypes that are distinguishable from wild-type (WT) α-synuclein.

These findings advance our understanding of how post-translational modifications of α-synuclein impact its function at synapses with important implications for disease pathogenesis.

## INTRODUCTION

α-Synuclein is a small, 140 amino acid protein that is expressed in neurons and enriched at synapses via interactions with synaptic vesicles (SVs) (Maroteaux *et al.*, 1988). Under normal, physiological conditions, α-synuclein functions in several steps of SV trafficking, including exocytosis, early stages of clathrin-mediated endocytosis (CME), and SV clustering/reclustering (Burre *et al.*, 2010; Vargas *et al.*, 2014, 2017; Logan *et al.*, 2017; Fouke *et al.*, 2021; Sharma and Burre, 2023). Although the molecular mechanisms are still under investigation, α-synuclein appears to cooperate with the synaptic phosphoprotein, synapsin, to regulate SV clustering, and vesicle recycling (Atias *et al.*, 2019; Hoffmann *et al.*, 2021; Stavsky *et al.*, 2023). α-Synuclein also interacts with the SNARE protein VAMP2 thereby regulating SNARE complex formation (Burre *et al.*, 2010; Diao *et al.*, 2013; Burre *et al.*, 2014). In Parkinson’s disease (PD) and dementia with Lewy bodies (DLB), α-synuclein aberrantly accumulates and aggregates throughout neurons, including at synapses (Spillantini *et al.*, 1998; Kramer and Schulz-Schaeffer, 2007; Schulz-Schaeffer, 2010). Such pathological accumulation of α-synuclein at synapses often correlates with cognitive deficits and dementia in PD and DLB, as well as some patients with Alzheimer’s disease (AD) (Schulz-Schaeffer, 2010; Sulzer and Edwards, 2019). In DLB, up to 40–90% of the aggregated α-synuclein may occur in the presynaptic compartment and precede Lewy body formation or other disease pathologies (Kramer and Schulz-Schaeffer, 2007; Schulz-Schaeffer, 2010). Thus, it is vitally important to understand how excess α-synuclein affects synapses.

Current evidence indicates that synaptic accumulation of α-synuclein severely impairs SV trafficking. We previously reported that acutely increasing wild-type (WT) α-synuclein levels at lamprey giant reticulospinal (RS) synapses inhibited clathrin-mediated and bulk endocytosis during high frequency stimulation (Clayton *et al.*, 2007; Cousin, 2009; Saheki and De Camilli, 2012; Chanaday *et al.*, 2019), which resulted in a severe loss of SVs, compensated by a build-up of plasma membrane and endocytic intermediates including clathrin-coated pits and vesicles (CCP/Vs) and bulk endosomes (Busch *et al.*, 2014; Medeiros *et al.*, 2017; Banks *et al.*, 2020; Soll *et al.*, 2020). Similarly, at the mammalian calyx of Held, acute dialysis of recombinant human α-synuclein into the presynaptic terminal also inhibited endocytosis and vesicle replenishment during high frequency stimulation with no apparent effects on either exocytosis or basal synaptic transmission (Xu *et al.*, 2016; Eguchi *et al.*, 2017). Likewise, overexpression of α-synuclein in mammalian hippocampal or dopaminergic neurons also inhibited SV trafficking, including SV reclustering, and induced a compensatory reduction in synapsin expression (Nemani *et al.*, 2010; Scott *et al.*, 2010; Scott and Roy, 2012). Overexpression of α-synuclein, as well as endocytic uptake of α-synuclein fibrils, induce synaptic pathologies that precede Lewy body formation (Volpicelli-Daley *et al.*, 2011; Boassa *et al.*, 2013). Thus, data from multiple synapse models are in agreement that excess α-synuclein is deleterious to SV trafficking, predominantly during SV recycling. However, the underlying molecular mechanisms are still unknown, including potential roles for α-synuclein post-translational modifications (PTMs).

Numerous PTMs have been reported for α-synuclein, including multiple phosphorylation, glycosylation, ubiquitination, and nitration sites, which when modified, substantially alter its membrane binding and aggregation properties in vitro (Paleologou *et al.*, 2008, 2010; Oueslati *et al.*, 2012; Zhang *et al.*, 2019; Galesic *et al.*, 2021; Balana *et al.*, 2023). Of particular interest is the phosphorylation of α-synuclein at serine 129 (pS129). Recent studies have reported that α-synuclein is reversibly phosphorylated at serine 129 by polo-like kinase 2 (PLK2) upon neuronal activity, a physiological event that regulates its interactions with synaptic binding partners, as well as synaptic transmission and plasticity (Mbefo *et al.*, 2010; Weston *et al.*, 2021b; Parra-Rivas *et al.*, 2022; Ramalingam *et al.*, 2023). While pS129 comprises only ∼4% of the total α-synuclein under normal conditions, in PD, DLB, and other synucleinopathies, pS129 α-synuclein greatly accumulates in Lewy bodies and neurites (Fujiwara *et al.*, 2002; Anderson *et al.*, 2006). Remarkably, up to 90% of aggregated α-synuclein in DLB brains contains the pS129 modification (Fujiwara *et al.*, 2002), and this is the predominant PTM found in DLB Lewy bodies (Anderson *et al.*, 2006). pS129 α-synuclein also accumulates at synapses in DLB brains, where it accounts for ∼20–25% of total pS129 deposits (Colom-Cadena *et al.*, 2017). In mouse and zebrafish models overexpressing α-synuclein, pS129 is also present in synaptic accumulations of α-synuclein (Scott *et al.*, 2010; Spinelli *et al.*, 2014; Weston *et al.*, 2021a). Although pS129 α-synuclein is traditionally thought of as a pathological biomarker for aggregated α-synuclein in Lewy bodies and neurites (Oueslati, 2016), the pS129 modification alone does not promote aggregation at synapses, indicating some other undetermined impacts of this modification (Weston *et al.*, 2021a). A recent study suggested that the S129 α-synuclein phosphomimic (S129D mutation) enhanced the SV trafficking deficits caused by α-synuclein overexpression (Parra-Rivas *et al.*, 2022), however the precise impacts and underlying mechanisms remain unclear.

The lamprey RS synapse is an ideal model in which to study the impacts of pS129 α-synuclein on synapse structure and function because we can acutely deliver precise amounts of recombinant pS129 α-synuclein directly to the presynaptic compartment via axonal microinjection, followed by controlled stimulation conditions and detailed image analysis to determine the impacts on vesicle trafficking. We have already used the lamprey RS synapse model to investigate how excess WT α-synuclein affects SV trafficking, as well as multiple PD-associated mutations and disease variants, providing a solid foundation against which to interpret the new findings (Busch *et al.*, 2014; Soll *et al.*, 2020). Moreover, the ability to introduce recombinant pS129 α-synuclein provides a distinct advantage over the standard expression of phosphomimics (S129E and S129D), because the phosphomimics do not recapitulate many of the biochemical characteristics of the phosphorylated protein, including the extended structure and in vitro aggregation properties (Paleologou *et al.*, 2008). Using this approach, we report here that, compared with WT α-synuclein, pS129 enhanced α-synuclein’s ability to bind synaptic membranes in vitro and also increased microclustering of SVs. When introduced acutely to lamprey synapses, excess pS129 α-synuclein also caused activity-dependent SV trafficking defects in vivo that were distinct from WT α-synuclein*,* including a greater amount of SV declustering, as well as dispersion of SVs into smaller microclusters. Thus, pS129 α-synuclein induces measurable and significant impacts on SV dynamics and vesicle trafficking, emphasizing the functional importance of α-synuclein PTMs while also providing novel insights into the mechanisms of synaptic pathologies in PD, DLB, and other synucleinopathies.

## RESULTS

### Characterization of recombinant WT and pS129 α-synuclein

**FIGURE 1:**
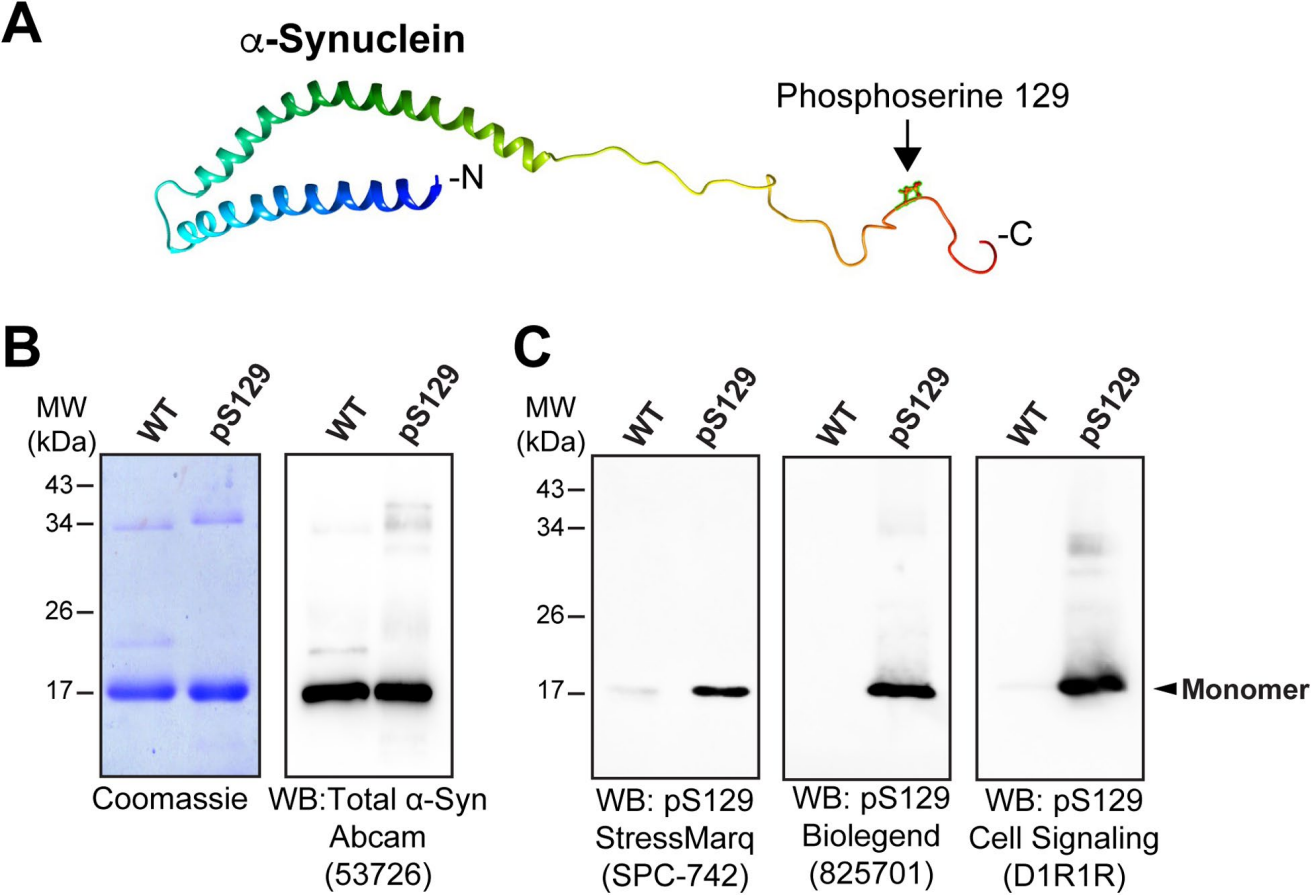
Characterization of pS129 α-synuclein. (A) NMR structure of human α-synuclein showing the pS129 modification. Ribbon model was generated in UCSF Chimera 1.16 using the UniProt PDB file: 1XQ8 of lipid micelle-bound α-synuclein (Ulmer *et al.*, 2005). (B) Coomassie gel (left; 2 μg/lane) and Western blot (right; 0.2 μg/lane) on recombinant WT and phosphoserine129-modified (pS129) human α-synuclein. Both proteins ran on a 12% SDS–PAGE primarily in monomeric form at ∼17 kDa. Western blot for total α-synuclein was performed using a pan-synuclein antibody that recognizes a common epitope in the N-terminal domain. (C) Western blots using pS129-specific α-synuclein antibodies confirmed the identity and stability of pS129 α-synuclein. Each lane contained either 250 ng (left blot) or 500 ng (middle and right blots) of protein.

Human α-synuclein binds avidly to small vesicles such as SVs. The NMR structure of α-synuclein bound to lipid micelles has been solved (Ulmer and Bax, 2005; Ulmer *et al.*, 2005). Lipid bound α-synuclein contains: an N-terminal lipid binding region that folds into a broken amphipathic alpha-helix in the presence of highly curved vesicles (a.a. 1-60); a nonamyloid component that regulates α-synuclein self-association (a.a. 61-95); and an intrinsically disordered, acidic C-terminal region that modulates vesicle binding (a.a. 96-140) (Davidson *et al.*, 1998; Chandra *et al.*, 2003; Burre *et al.*, 2014; Lautenschlager *et al.*, 2018). The phosphorylation of serine 129 by PLK2 occurs near the C-terminus of α-synuclein (Figure 1A). We obtained recombinant human pS129 α-synuclein from Proteos (Kalamazoo, MI). This protein was generated via native chemical ligation of a purified, *Escherichia coli (E. coli)*-generated recombinant fragment of α-synuclein (a.a. 1-84) and a synthetic phosphopeptide (a.a. 85-140). WT α-synuclein was obtained from rPeptide (Watkinsville, GA). Both WT and pS129 α-synuclein ran as monomers at ∼17 kDa on SDS–PAGE, as shown by Coomassie staining and Western blotting for total α-synuclein, though a minor fraction of dimers around 34 kDa is often present (Figure 1B). Western blotting with three different pS129-specific α-synuclein antibodies confirmed the identity of the recombinant protein and stability of the phosphate modification (Figure 1C).

### WT and pS129 α-synuclein preferentially bind to membrane lipids enriched at synapses

**FIGURE 2:**
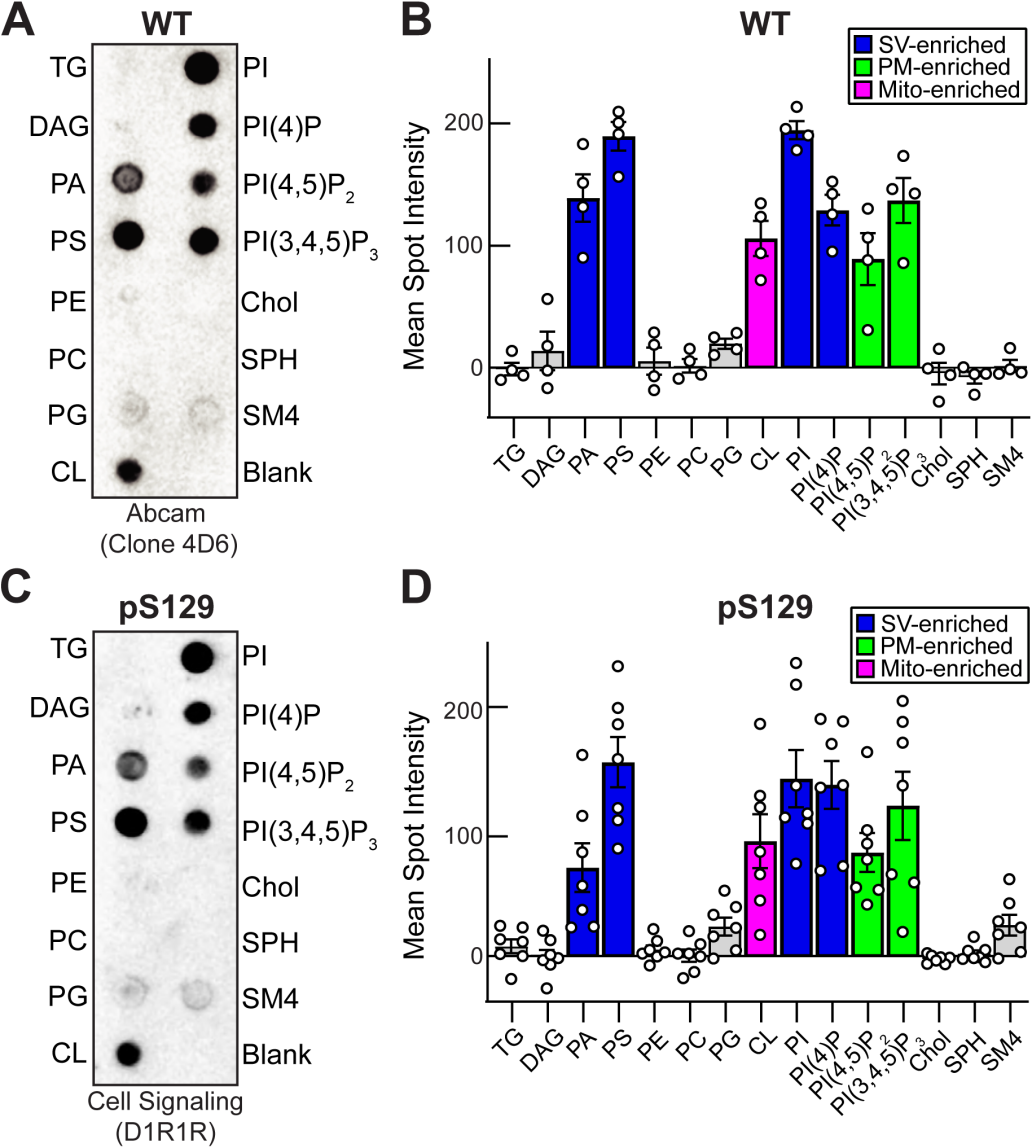
WT and pS129 α-synuclein preferentially bind membrane lipids enriched at synapses. (A) Protein-lipid overlay showing that WT α-synuclein bound preferentially to PA, PS, PI, PI(4)P, PI(4,5)P_2_, and PI(3,4,5)P_3_, as well as mitochondrial CL. WT α-synuclein was detected with a mouse monoclonal antibody (Abcam; ab1903 [clone 4D6]) (B) Quantification showing the lipid binding distribution for WT α-synuclein. Bars indicate mean ± SEM from *n* = 4 experiments and are colored according to their enrichment in SVs, blue; PM, green; or mitochondria (Mito; magenta). (C and D) pS129 α-synuclein exhibited a similar lipid binding profile to WT α-synuclein. pS129 α-synuclein was detected with a rabbit monoclonal (D1R1R; Cell Signaling). Bars indicate mean ± SEM from *n* = 7 experiments. TG, triglyceride; DAG, diacylglycerol; PA, phosphatidic acid; PS, phosphatidylserine; PC, phosphatidylethanolamine; PG, phosphatidylglycerol; CL, cardiolipin; PI, phosphatidylinositol; PI(4)P, phosphatidylinositol 4-phosphate; PI(4,5)P_2_, phosphatidylinositol 4,5-bisphosphate; PI(3,4,5)P_3_, phosphatidylinositol 3,4,5-trisphosphate; Chol, cholesterol; SPH, sphingomyelin; SM4, 3-sulfogalactosylceramide.

We first wanted to determine the lipid binding profile for pS129 α-synuclein in comparison to WT α-synuclein. Prior studies have shown that WT α-synuclein binds avidly to anionic lipids within highly curved liposomes in vitro (Burre *et al.*, 2010, 2012; Pranke *et al.*, 2011; Busch *et al.*, 2014). These include phosphatidic acid (PA), phosphatidylserine (PS), phosphatidylinositol (PI), and phosphatidylinositol-4-phosphate (PI[4]P), which are enriched in SVs, as well as phosphatidylinositol-4,5-bisphosphate (PI[4,5]P_2_), and phosphatidylinositol-3,4,5-triphosphate (PI[3,4,5]P_3_), which are enriched in the PM (Davidson *et al.*, 1998; Burre *et al.*, 2012; Soll *et al.*, 2020). Here, we tested an expanded array of phospholipids in a protein-lipid overlay assay using Membrane Lipid Strips^TM^, which were spotted with 15 different lipids commonly found in cell membranes (Echelon Biosciences; Salt Lake City, UT). Membrane Lipid Strips were incubated with either 10 or 20 μg/ml recombinant WT or pS129 α-synuclein, after which any bound protein was detected with WT- or pS129-specific antibodies using enhanced chemiluminescence (ECL). As expected, WT α-synuclein bound primarily to anionic lipids enriched on SVs (PA, PS, PI, PI[4]P) and plasma membrane (PI[4,5]P_2_, PI[3,4,5]P_3_), but not to neutral phospholipids such as phosphatidyl ethanolamine (PE) or phosphatidylcholine (PC) (Figure 2, A and B) (Davidson *et al.*, 1998; Burre *et al.*, 2012; Busch *et al.*, 2014). WT α-synuclein also bound to cardiolipin (CL), which is synthesized in the inner mitochondrial membrane. In contrast, very little binding was observed for triglycerides (TG), diacylglycerol (DAG), cholesterol (Chol), sphingomyelin (SPH), and 3-sulfogalactosylceramide (SM4) (Figure 2, A and B). The lipid binding profile for pS129 α-synuclein was nearly identical to that for WT α-synuclein (Figure 2, C and D). Thus, both WT and pS129 α-synuclein preferentially bind to anionic phospholipids enriched at synapses and in mitochondria with negligible differences between their overall lipid binding profiles, as expected since their N-terminal domains are identical.

### pS129 exhibits enhanced binding and oligomerization on synaptic membranes in vitro

**FIGURE 3:**
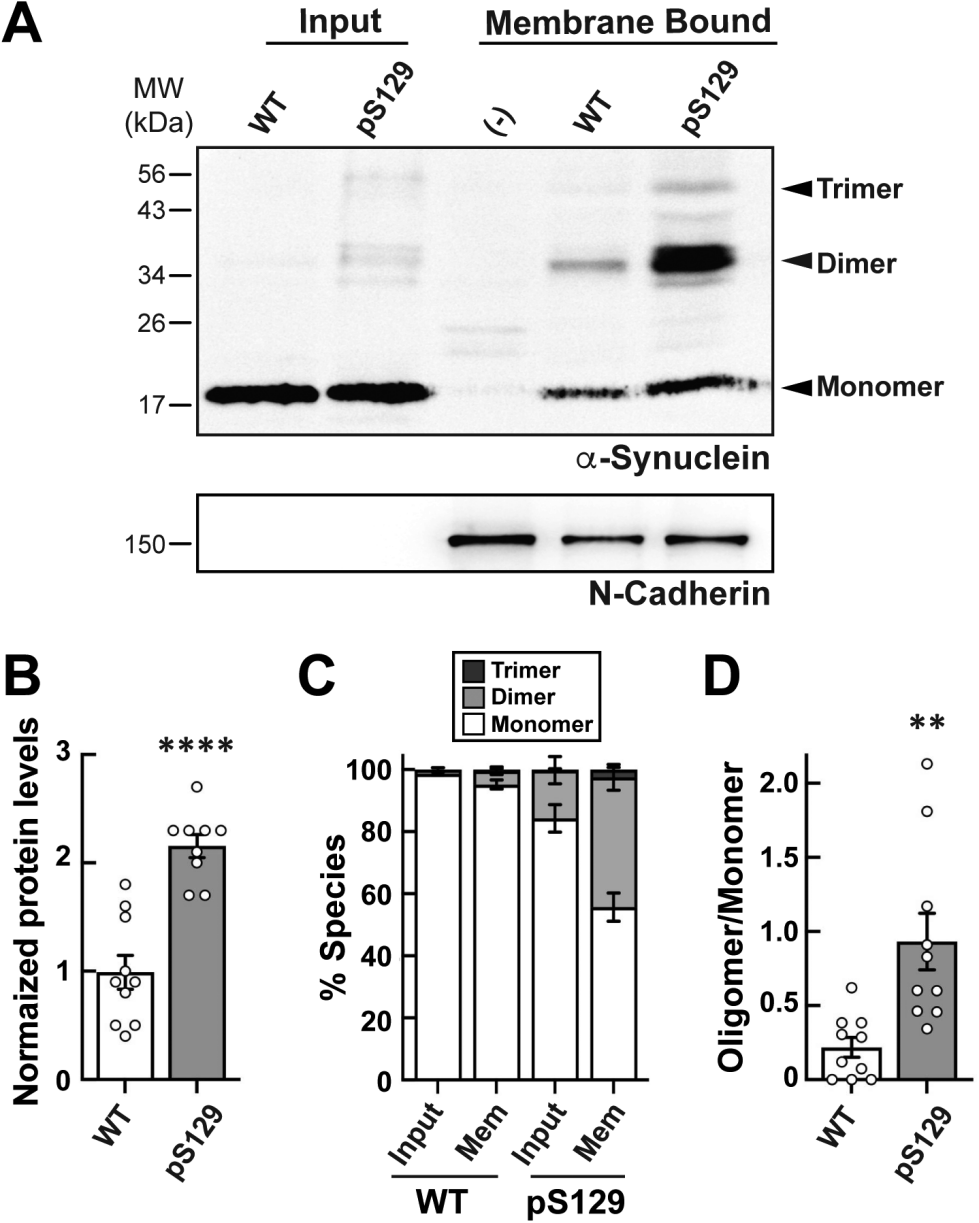
Enhanced binding and oligomerization of pS129 α-synuclein on synaptic membranes. (A) Isolated membranes from mouse brain synaptosomes were incubated with 2 μM WT or pS129 α-synuclein. After washing, any bound WT or pS129 was detected by Western blotting using a pan-synuclein antibody (Abcam 53726). While WT α-synuclein was readily recruited to synaptic membranes, pS129 showed enhanced binding and oligomerization into dimers and trimers. N-cadherin was used as a loading control for the membranes. (–) indicates membranes that were incubated with cytosol but not supplemented with WT or pS129. (B) Band intensity analysis revealed a 2.2-fold increase in pS129 recruitment to synaptic membranes, compared with WT α-synuclein. (C and D) Oligomerization of pS129 on synaptic membranes was also greater than that observed for WT α-synuclein. Bars in B-D indicate mean ± SEM from *n* = 9–10 experiments; ** indicates *p* < 0.005 and **** indicates *p* < 0.0001 by Student’s *t* test.

We next wanted to test whether the pS129 modification alters α-synuclein’s binding to purified synaptic membranes, as the synaptic phenotypes and cellular toxicity induced by excess α-synuclein are highly correlated with its membrane binding properties (Nemani *et al.*, 2010; Busch *et al.*, 2014; Wang *et al.*, 2014; Dettmer *et al.*, 2017; Kim *et al.*, 2021). We therefore performed an in vitro membrane binding assay using purified synaptosomal membranes from mouse brains, which comprise a more complex, physiological lipid composition (Soll *et al.*, 2020; Vargas *et al.*, 2021). Synaptosomal membranes were isolated and stripped of associated proteins, leaving only transmembrane proteins like N-cadherin. The stripped membranes were then incubated with brain cytosol supplemented with 2 μM recombinant WT or pS129 α-synuclein, followed by Western blotting for total α-synuclein to determine the extent of α-synuclein binding to synaptic membranes. While both WT and pS129 α-synuclein bound readily to purified synaptic membranes, pS129 exhibited stronger binding and greater oligomerization (Figure 3). WT α-synuclein remained predominantly in monomeric form, however pS129 further oligomerized into dimers and trimers (Figure 3A). Quantitative analysis of the band intensities revealed a greater than twofold increase of α-synuclein binding to synaptic membranes when phosphorylated at serine 129 (Figure 3B; WT: 0.99 ± 0.16; pS129: 2.16 ± 0.11; *n* = 9 and 10; *p* < 0.0005; unpaired Student’s *t* test). Moreover, while only ∼15% of WT α-synuclein oligomerized into higher molecular weight species upon membrane binding, nearly 50% of pS129 α-synuclein oligomerized into dimers and trimers (Figure 3C). This resulted in a 4.2-fold increase in the oligomer to monomer ratio with pS129 α-synuclein, compared with WT (Figure 3D; WT oligomer/monomer: 0.22 ± 0.07; pS129 oligomer/monomer: 0.93 ± 0.19, *n* = 10; *p* = 0.0024; unpaired Student’s *t* test). We confirmed that WT α-synuclein did not become phosphorylated at serine 129 upon binding to synaptic membranes under these conditions (Supplemental Figure S1). Thus, pS129 α-synuclein exhibits stronger binding and oligomerization on synaptic membranes in vitro than its WT counterpart, suggesting that it would also produce distinct effects on synapses in vivo.

### pS129 modification enhances α-synuclein-mediated SV “microclustering” in vitro

The enhanced membrane binding and oligomerization observed with pS129 α-synuclein also prompted us to examine how this phosphorylation event impacts SV clustering. α-Synuclein alone can induce SVs to form “microclusters” in vitro (Diao *et al.*, 2013; Aryal *et al.*, 2021; Hoffmann *et al.*, 2021). Synuclein also regulates SV clustering and reclustering in vivo at lamprey and mammalian synapses, respectively (Fortin *et al.*, 2005; Nemani *et al.*, 2010; Vargas *et al.*, 2017; Fouke *et al.*, 2021). Moreover, α-synuclein interacts with synapsin I, an abundant SV-associated phosphoprotein that clusters SVs and regulates the distal reserve pool of vesicles (De Camilli *et al.*, 1983a, 1983b; Pieribone *et al.*, 1995; Zhang and Augustine, 2021). Overexpression of α-synuclein causes a compensatory reduction in synapsin expression (Nemani *et al.*, 2010; Scott *et al.*, 2010), and vice versa, triple knockout of α/β/γ-synucleins increases synapsin expression (Greten-Harrison *et al.*, 2010), indicating an interaction between α-synuclein and synapsin that has also been confirmed using functional and biochemical approaches (Atias *et al.*, 2019; Hoffmann *et al.*, 2021; Stavsky *et al.*, 2023). We therefore tested the behavior of pS129 α-synuclein in an established in vitro vesicle clustering assay with and without synapsin present.

**FIGURE 4:**
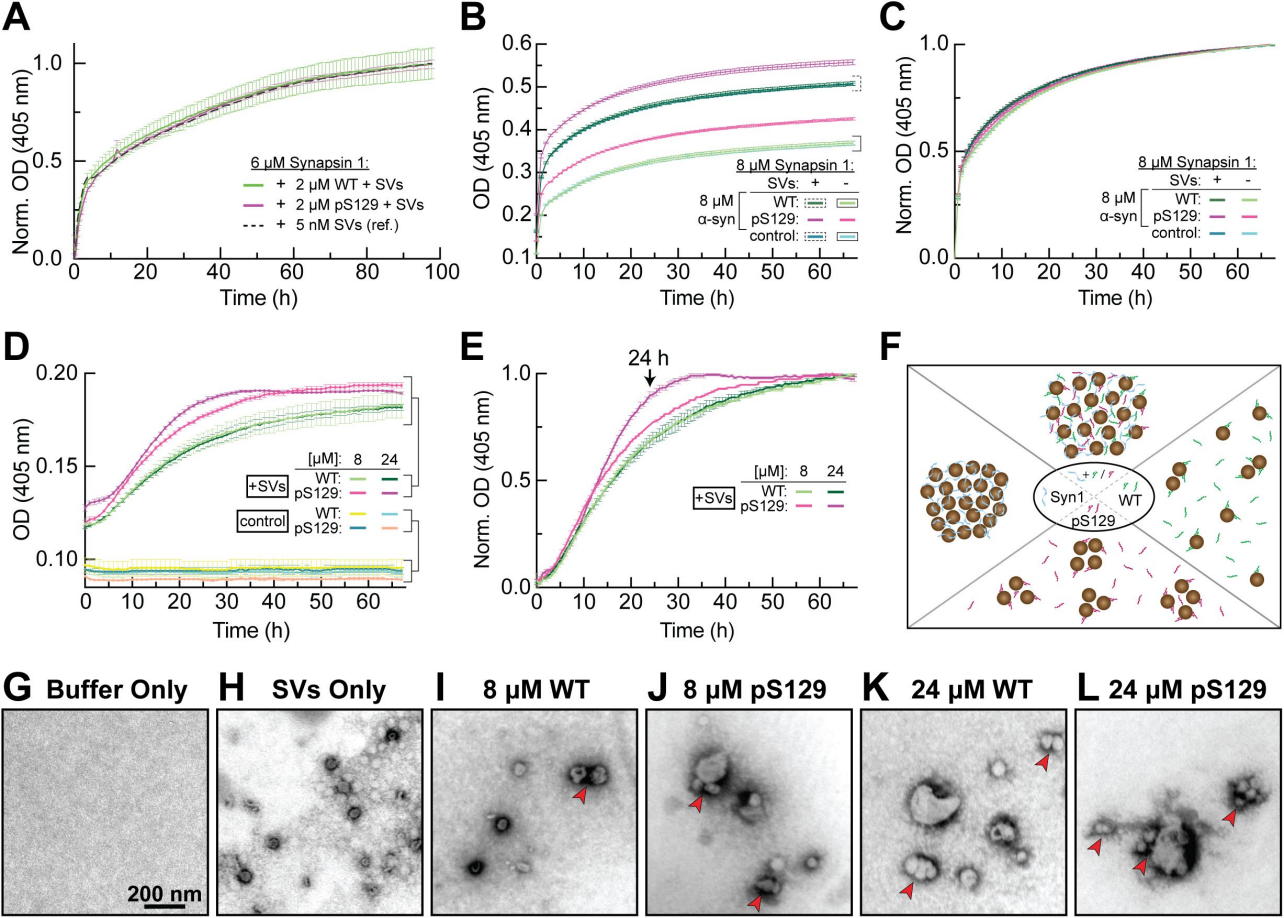
pS129 enhances α-synuclein-induced SV “microclustering” in vitro. (A) Synapsin I (6 μM) robustly clustered SVs in vitro, as shown by the rapid, biphasic increase in turbidity at OD 405 nm (black reference curve). Addition of 2 μM WT or pS129 α-synuclein, maintaining the physiological 3:1 M ratio of synapsin 1: α-synuclein, had little impact on synapsin-SV condensation under these conditions. Data points represent mean ± SEM from *n* = 4 experiments. (B and C) Even when synapsin 1 and α-synuclein were in equimolar ratios (8 μM for each), α-synuclein had little impact on the rate of synapsin-SV condensation. (D) In the absence of synapsin, both 8 μM (*n* = 2) and 24 μM (*n* = 4) WT and pS129 α-synuclein induced moderate SV clustering in vitro, however pS129 produced greater, dose-dependent effects. Control curves show results with WT and pS129 α-synuclein protein alone, in the absence of SVs, demonstrating that the increased turbidity is due to SV microclustering. (E) Normalized data from panel D. Arrow indicates the 24-h time point from which samples were imaged (see [G–L]). (F) Working model illustrating how WT and pS129 α-synuclein could impact SV microclustering and synapsin-SV clustering (see also Hoffmann *et al.*, 2021). (G–L) EM images (37,000x) showing SV microclustering induced by α-synuclein. At the beginning of the incubation period, purified SVs were generally monodispersed and appeared as single vesicles (H). After 24 h of incubation with either 8 or 24 μM WT α-synuclein or pS129 α-synuclein, small SV microclusters were visible (arrows in I–L). In both cases, pS129 enhanced SV microclustering. Scale in (G) applies to (H–L).

At physiological concentrations, synapsin I forms distinct condensates via liquid-liquid phase separation (LLPS), which can recruit both SVs and α-synuclein (Milovanovic and De Camilli, 2017; Milovanovic *et al.*, 2018; Hoffmann *et al.*, 2021). However, synapsin is the main driver of LLPS (Hoffmann *et al.*, 2021). As previously reported, combining 6 μM synapsin I with SVs purified from adult rat brains resulted in robust SV clustering in vitro, shown by a biphasic increase in turbidity measured at OD 405 nm (Figure 4A). Addition of 2 μM WT or pS129 α-synuclein (Supplemental Figure S2), mimicking the physiological molar ratio of synapsin:α-synuclein (3:1) (Wilhelm *et al.*, 2014), had little effect on synapsin-SV condensation, confirming that synapsin I predominates the LLPS reaction under these conditions (Figure 4A). When repeated with an equimolar ratio of synapsin:α-synuclein (1:1) at 8 μM, although the pS129 curves started at a higher level, the normalized data also showed no difference in the turbidity kinetics after adding WT or pS129 α-synuclein, once again confirming synapsin I as the main driver for the LLPS reaction (Figure 4, B and C) (Hoffmann *et al.*, 2021).

Given that α-synuclein alone can cross-link small vesicles into “microclusters” (Diao *et al.*, 2013; Lautenschlager *et al.*, 2018; Aryal *et al.*, 2021), we set out to determine whether the pS129 modification would alter these microclusters. We therefore repeated the experiments with only SVs in the presence of 8 and 24 μM α-synuclein (WT or pS129), covering the range of physiological concentrations of WT α-synuclein at synapses, which has been reported between ∼3–6 μM and up to 40 μM (Westphal and Chandra, 2013; Wilhelm *et al.*, 2014). Synapsin I was not included in these experiments. Under these conditions, WT α-synuclein (8 and 24 μM) induced a moderate amount of SV clustering in vitro with little difference in the time courses (Figure 4, D and E). In contrast, pS129 (8 and 24 μM) enhanced SV clustering in vitro in a dose-dependent manner (Figure 4, D and E). Thus, consistent with the increased membrane binding and oligomerization, the pS129 modification also enhanced SV microclustering in vitro (Figure 4F). To confirm the presence of the α-synuclein-induced SV microclusters, we imaged the samples from the turbidity assays using electron microscopy, focusing on the 24-h time point where the differences between WT and pS129 were most prominent. EM imaging confirmed that both WT and pS129 α-synuclein induced a dose-dependent microclustering of SVs and that the pS129 modification further enhanced this SV microclustering effect (Figure 4, G–L). At higher concentrations of pS129 especially, the SV microclusters were larger and more heterogeneous (Figure 4L). Taken together, these results predict that pS129 will also impact SV clustering in vivo.

### pS129 α-synuclein localizes to SV clusters within lamprey giant axons

**FIGURE 5:**
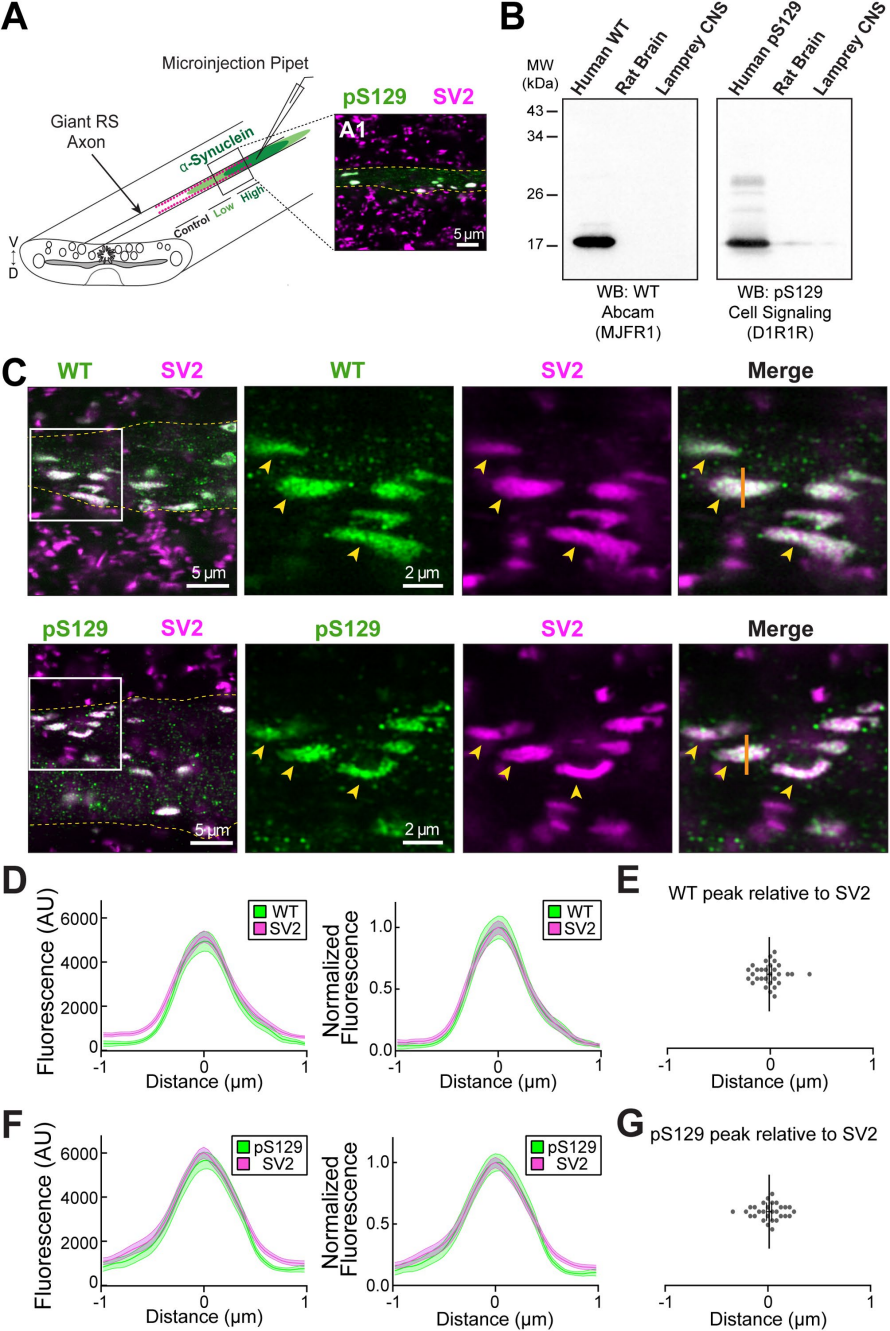
pS129 and WT α-synuclein localize to SV clusters in lamprey axons. (A) Diagram showing the microinjection strategy for α-synuclein into lamprey giant RS axons. (A1) Inset shows a small region of a whole mounted lamprey spinal cord immunostained for the injected α-synuclein (pS129) and endogenous SV2, a marker of SV clusters. Dotted lines denote the borders of a single giant RS axon. (B) Characterization of the WT and pS129-specific α-synuclein antibodies used for IF experiments. Western blotting confirms the antibody is specific for human α-synuclein with little or no cross-reactivity against endogenous synuclein in rat brain or lamprey CNS. Antibodies used are as indicated. (C) Giant RS axons were injected with recombinant WT or pS129 α-synuclein and subsequently immunostained for α-synuclein (green) and SV2 (magenta). High resolution confocal images were obtained using a Zeiss LSM980 Axio Examiner with Airyscan2 (63X, 1.4 NA objective). Dotted lines indicate border of injected axon. White box in leftmost panel indicates the ROIs for the subsequent images. Arrows indicate SV clusters colabeled with α-synuclein and SV2. Solid line in the rightmost merged image indicates the synaptic position from which the intensity analysis was performed. (D) Histogram analysis of both raw (left) and normalized (right) fluorescence intensities showed a strong colocalization between injected WT α-synuclein and the SV2-positive SV clusters. (E) Peak analysis confirmed the strong colocalization between WT α-synuclein and SV2. (F and G) pS129 also colocalized to SV clusters. Data were obtained from *n* = 29-30 synapses, four to five axons per condition.

We next wanted to test how pS129 α-synuclein impacts SV trafficking and clustering in vivo at lamprey RS synapses. For these experiments, we microinjected recombinant WT or pS129 α-synuclein into lamprey giant RS axons, which delivers the proteins directly to presynapses (Figure 5A). The first step was to determine the extent to which axonally-injected recombinant human α-synuclein interacts with SV clusters in vivo. We therefore performed whole mount immunofluorescence (IF) on lamprey spinal cords and probed for the injected human α-synuclein, as well as SV glycoprotein 2 (SV2), a marker for SV clusters in all vertebrates tested, including lampreys (Figure 5, A1) (Buckley and Kelly, 1985; Busch and Morgan, 2012; Banks *et al.*, 2020). The antibodies used for detecting the injected WT and pS129 were specific for human α-synuclein and had minimal cross-reactivity with either rat or lamprey synucleins, as shown by Western blotting (Figure 5B) and IF (Supplemental Figure S3), thereby allowing us to selectively visualize the injected human α-synuclein proteins. After injection into unstimulated lamprey axons, both recombinant WT and pS129 α-synuclein bound strongly to SV clusters in vivo, as determined by colocalization with SV2 (Figure 5C). A histogram intensity analysis revealed a tight overlap between the SV2 and WT α-synuclein peak intensities and overall profile structure, with the WT peak being within error of the SV2 peak (Figure 5, D and E; WT: 0.085 ± 0.0235 μm, *n* = 30 synapses, *n* = 5 axons, three animals). Similarly, pS129 was also tightly colocalized and overlapping with SV2 at presynapses (Figure 5, C, F and G; pS129: 0.013 ± 0.025 μm, *n* = 29 synapses, *n* = 4 axons, three animals). A Manders’ colocalization coefficient (MCC) analysis confirmed that both WT and pS129 α-synuclein (green fluorescence signal) were highly colocalized with SV2 (red fluorescence signal) on SV clusters (WT: MCC_α-Syn:SV2_ = 0.924 ± 0.022, MCC_SV2:α-Syn_ = 0.827 ± 0.031, *n* = 29 ROIs, *n* = 5 axons, three animals; pS129: MCC_α-Syn:SV2_ = 0.904 ± 0.022, MCC_SV2:α-Syn_ = 0.868 ± 0.030, *n* = 4 axons, three animals). Furthermore, the density of α-synuclein–associated fluorescence that was localized at synapses was significantly higher than in the surrounding area with only 15–24% being detected in the nonsynaptic axoplasm (WT_[synapse]_: 12,794 ± 2181 AU/μm^2^; WT_[axoplasm]_: 1834 ± 403 AU/μm^2^, WT_[axoplasm:synapse]_: 15.3% ± 2.3%, *n* = 10 images, *n* = 5 axons, three animals; pS129_[synapse]_: 11,355 ± 1520 AU/μm^2^; pS129_(axoplasm)_: 2480 ± 216 AU/μm^2^, pS129_[axoplasm:synapse]_: 24.3% ± 2.6%; *n* = 11 images, *n* = 4 axons, three animals). We did not detect any obvious differences in the localization patterns for the injected WT versus pS129 α-synuclein. Thus, upon injection, both WT and pS129 α-synuclein localize in vivo at the large SV clusters within lamprey giant axons, as is to be expected for this SV-associated protein. The data also suggest that the SV cluster is likely to be the main target of any synaptic effects caused by excess α-synuclein.

### pS129 α-synuclein has little effect on unstimulated lamprey synapses

**FIGURE 6:**
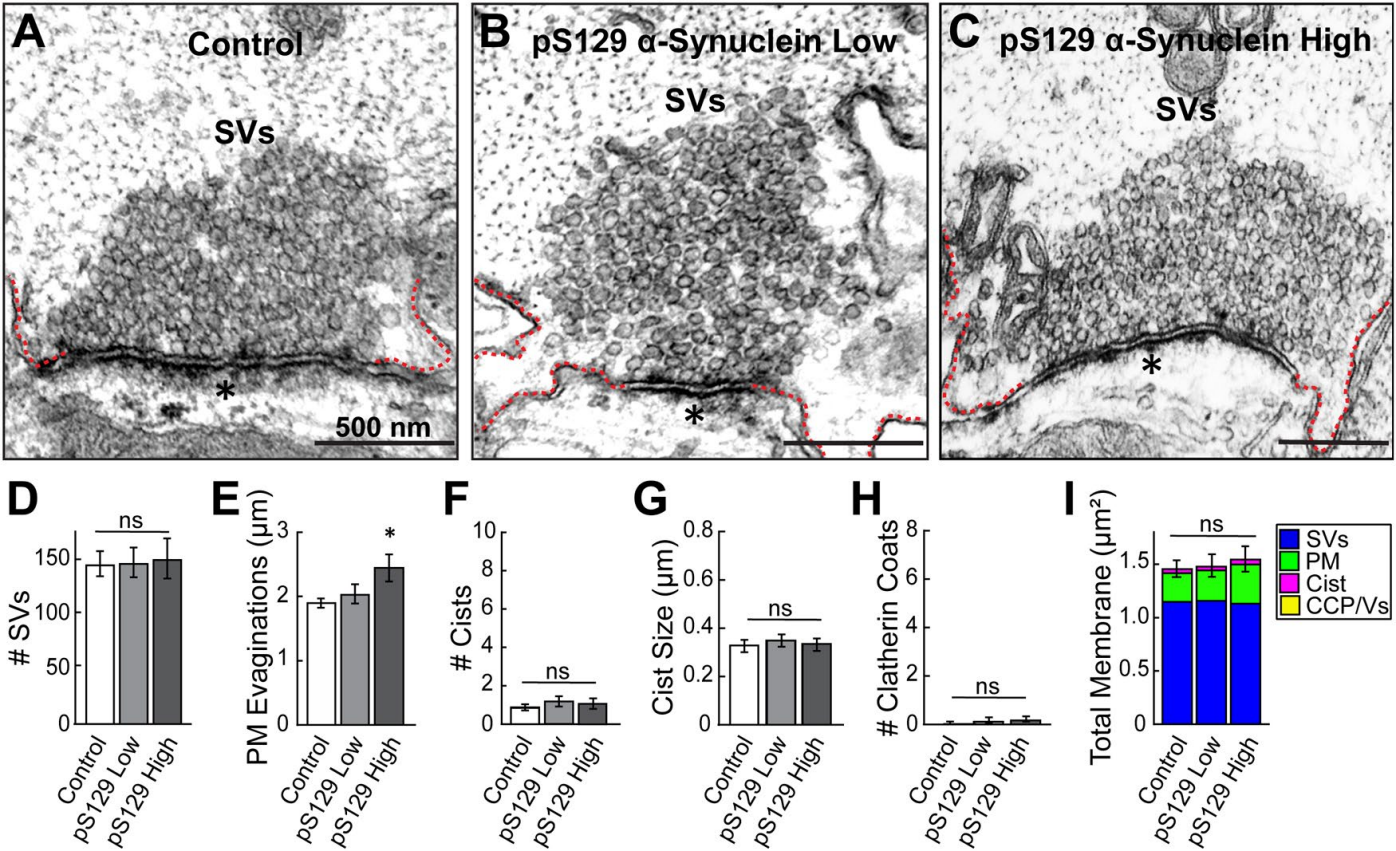
Excess pS129 α-synuclein does not affect the SV clusters at resting lamprey synapses. (A) Electron micrograph of an unstimulated control lamprey synapse showing a large, densely-packed cluster of SVs. (B and C) After introducing low (5–8 μM) or high (10–20 μM) concentrations of pS129, there was no obvious effect on the SV clusters. Dotted lines indicate PM evaginations. Asterisk indicates postsynaptic density. Scale bar in A applies to B and C. (D–H) Quantification revealed no significant difference in the number of SVs, number and size of Cists, nor CCP/Vs (Clathrin Coats), at synapses treated with pS129. Only the PM evaginations were larger. (I) There was no change in the total synaptic membrane after treatment with pS129. Bars in D–I indicate mean ± SEM from *n* = 22–39 synapses, two axons/animals. “*ns”* indicates “not significant” and * indicates *p* < 0.05 by one-way ANOVA.

We previously reported that acute introduction of excess WT human α-synuclein or lamprey γ-synuclein into the lamprey giant RS axons had no obvious effect on the ultrastructure of SV clusters at unstimulated synapses or during low frequency stimulation (5 Hz) (Busch *et al.*, 2014). We therefore evaluated how excess pS129 α-synuclein impacts resting SV clusters. Coinjection of pS129 α-synuclein along with a fluorescent dye of similar molecular weight (AlexaFluor^TM^ 488 dextran, 10 kDa) allowed us to estimate the final concentration of pS129 within the axons. At regions closest to the injection site, the estimated concentration of pS129 was ∼10–20 μM (which we refer to as the “high” concentration; see Figure 5A). At regions farther from the injection site, the estimated concentration was ∼5–8 μM (which we refer to as the “low” concentration). These concentrations are 0.5–3.0x higher than the best estimates of α-synuclein concentrations at synapses and commensurate with mild to moderate α-synuclein overexpression in the brains of PD patients (Singleton *et al.*, 2003; Miller *et al.*, 2004; Westphal and Chandra, 2013; Wilhelm *et al.*, 2014). Images of untreated synapses were obtained from the same injected axons but at distances farther away, beyond the regions where the injected α-synuclein had diffused, thus providing an internal control. Acute introduction of pS129 α-synuclein to unstimulated axons had no major ultrastructural effects on resting synapses, as the SV clusters remained large and tightly packed around the active zone (AZ), similar to control synapses (Figure 6, A–C). Quantification of the SV numbers revealed no significant difference in the average size of the vesicle clusters upon introduction of low or high concentrations of pS129 α-synuclein (Figure 6D; Control: 145.5 ± 10.2 SVs/synapse; *n* = 39 synapses, two axons/animals; pS129 [low]: 146.9 ± 12.4 SVs/synapse; *n* = 23 synapses, two axons/animals; pS129 [high]: 150.5 ± 17.4 SVs/synapse; *n* = 22 synapses, two axons/animals; one-way ANOVA, *p* = 0.9600). We also measured the adjacent PM, presumptive endosomes (which we call “cisternae” [Cist]), and CCP/Vs (see *Materials and Methods*; Figure 6, E–H). This analysis revealed that excess pS129 α-synuclein induced no significant changes in the number or size of Cist, or CCP/Vs, however there was a modest increase in the size of the PM evaginations (Figure 6E; Control: 1.91 ± 0.07 μm/synapse; *n* = 39 synapses; pS129 [low]: 2.05 ± 0.15 μm/synapse; *n* = 23 synapses; pS129 [high]: 2.47 ± 0.21 μm/synapse; *n* = 22 synapses, two axons/animals; one-way ANOVA, *p* = 0.0140) Nonetheless, when combined together, there was no significant change in the total membrane area at synapses (Figure 6I) (Control: 1.46 ± 0.08 μm^2^/synapse; *n* = 39 synapses, two axons/animals; pS129 [low]: 1.49 ± 0.10 μm^2^/synapse; *n* = 23 synapses, two axons/animals; pS129 [high]: 1.56 ± 0.12 μm^2^/synapse; *n* = 22 synapses, two axons/animals; one-way ANOVA, *p* = 0.7794]. Thus, pS129 α-synuclein did not induce substantial effects on the morphology of resting lamprey synapses, only a slight increase in PM evaginations that may be related to its enhanced membrane binding.

### pS129 α-synuclein impairs SV trafficking at stimulated synapses, resulting in a severe depletion of SVs

**FIGURE 7:**
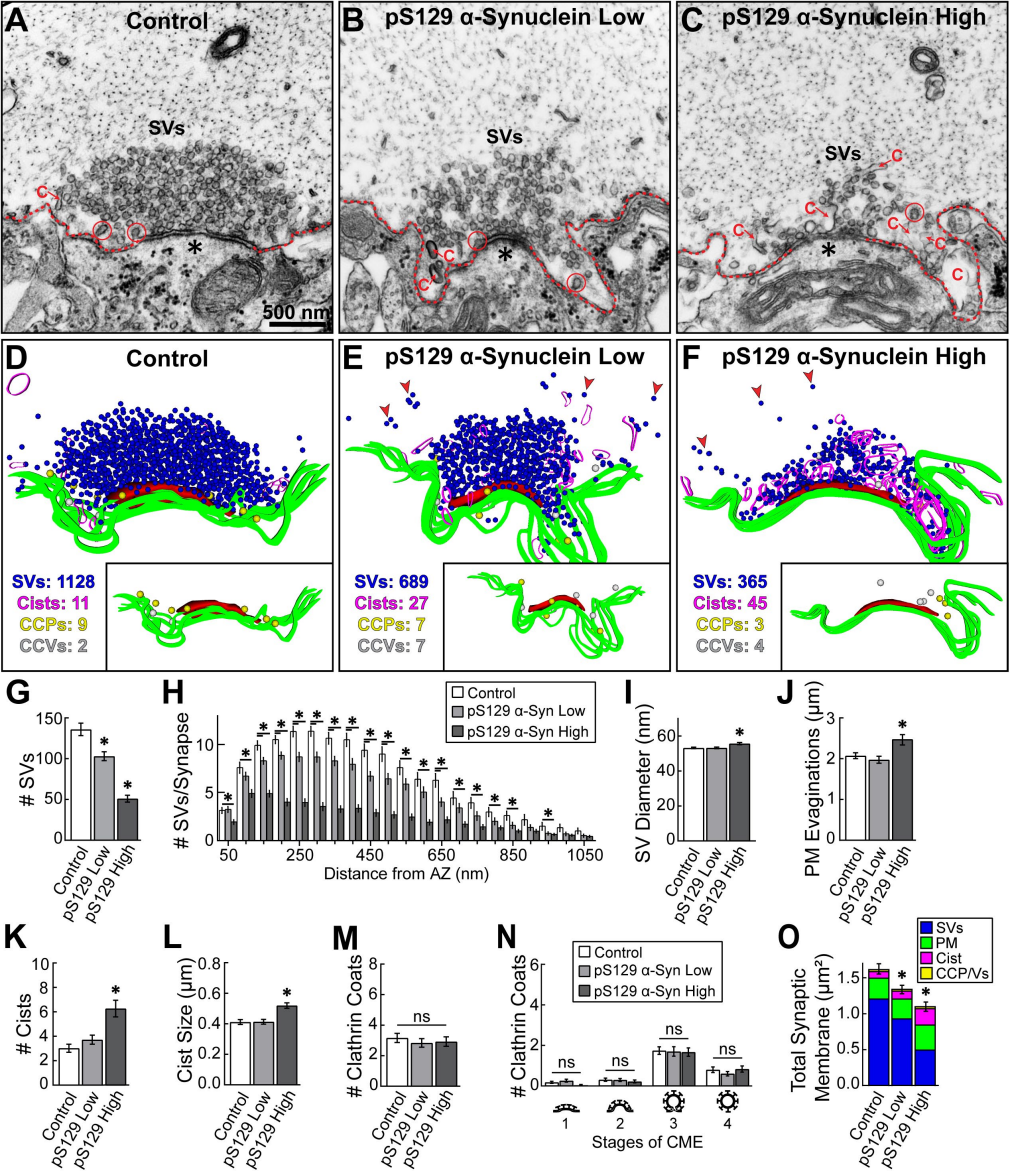
Excess pS129 α-synuclein causes a severe depletion of SVs at stimulated lamprey synapses. (A–C) Electron micrographs showing a progressive loss of SVs after introducing pS129 at low (5–8 μM) or high (10–20 μM) concentrations to stimulated lamprey RS synapses (20 Hz, 5 min). pS129 also caused a moderate expansion of the PM (dotted lines) and build-up of atypical vesicular “Cist” (C), which are likely endosomes. Circles indicate CCP/Vs. Asterisks indicate postsynaptic density. Scale bar in A applies to B and C. (D–F) three-dimensional reconstructions of the synapses from panels A–C. Blue spheres = SVs; magenta ribbons = Cist; yellow and white spheres = CCPs and CCVs, respectively. PM is indicated by green ribbons, and AZ is shown as a red slab. The loss of SVs and build-up of Cist are especially prominent. Insets show the CCP/V distribution at these synapses. Red arrows show several SV microclusters that appear to be dispersed from the main SV cluster. (G–I) Morphometric analysis revealed that pS129 caused a depletion of SVs at synapses and that the remaining SVs had a slightly larger diameter. (J–L) The loss of SVs was partially compensated by an expansion of the PM, as well as an increase in the number and size (perimeter) of Cist. (M–N) pS129 did not alter the number or distribution of CCP/Vs, unlike our previous studies on WT α-synuclein (Busch *et al.*, 2014; Medeiros *et al.*, 2017; Banks *et al.*, 2020). (O) A total membrane analysis revealed a significant loss of synaptic membrane, primarily due to SV depletion. Bars indicate mean ± SEM from *n* = 55–67 synapses, four axons/animals. * indicates *p* < 0.05, and “*ns”* indicates “not significant” by one-way ANOVA.

We previously reported that WT α-synuclein strongly inhibits SV recycling at lamprey synapses during high-frequency stimulation (20 Hz) with effects on both clathrin-mediated and bulk endocytosis (Busch *et al.*, 2014; Medeiros *et al.*, 2017, 2018; Banks *et al.*, 2020; Soll *et al.*, 2020). Specifically, there was an activity-dependent depletion of SVs, which was compensated by an expansion of the PM and aberrant build-up of endocytic intermediates including CCP/Vs and “Cist” (presumptive endosomes), with no loss in total membrane, indicating that synaptic membranes were simply redistributed and not escaping away from the local synaptic area. To assess how the pS129 modification impacts the effects of excess α-synuclein on SV trafficking, we examined synaptic ultrastructure at pS129-treated synapses after 20 Hz, 5 min stimulation. pS129 α-synuclein induced a severe depletion of SVs and a moderate increase in PM evaginations and Cist (Figure 7, A–F). Interestingly, at both low concentrations (∼5–8 μM) and high concentrations (∼10–20 μM) of pS129, some smaller microclusters of SVs containing only a few vesicles could be observed far away from the main vesicle cluster, suggesting altered SV clustering/reclustering dynamics during synaptic activity (Figure 7, D–F, red arrows). Quantification revealed a progressive, dose-dependent reduction in the number of SVs at synapses, reaching a 63% depletion of the vesicle pool at the high pS129 concentration (Figure 7G; Control: 135.9 ± 7.6 SVs/synapse; *n* = 55 synapses, four axons/animals; pS129 [low]: 103.1 ± 5.5 SVs; *n* = 61 synapses, four axons; pS129 [high]: 51.03 ± 4.17 SVs; *n* = 67 synapses, four axons; one-way ANOVA *p* < 0.0001). The depletion of SVs occurred throughout the vesicle cluster, both near to and far from the active zone (Figure 7H). The remaining SVs were slightly larger compared with those at control synapses (Figure 7I; Control: 53.20 ± 0.44 nm; pS129 [low]: 53.23 ± 0.48 nm; pS129 [high]: 55.65 ± 0.63 nm; *n* = 400 SVs, four axons; one-way ANOVA, *p* = 0.0026).

The PM evaginations were also somewhat enlarged at the high pS129 concentration (Figure 7J; Control: 2.07 ± 0.07 µm/synapse; *n* = 55 synapses, four axons; pS129 [low]: 1.98 ± 0.09 µm/synapse; *n* = 61 synapses, four axons; pS129 [high]: 2.48 ± 0.13 µm/synapse; *n* = 67 synapses, four axons; one-way ANOVA *p* = 0.0168). Moreover, there was a significant 2.1-fold increase in the number of Cist, as well as a 21% increase in their size (perimeter), compared with controls (Figure 7, K and L; # Cist - Control: 3.02 ± 0.33 Cist/synapse; *n* = 55 synapses, four axons; pS129 [low]: 3.72 ± 0.38 Cist/synapse; *n* = 61 synapses, four axons; pS129 [high]: 6.3 ± 0.7 Cist/synapse; *n* = 67 synapses, four axons; one-way ANOVA *p* < 0.0001; Cist Size - Control: 0.41 ± 0.02 µm/synapse; *n* = 55 synapses, four axons; pS129 [low]: 0.41 ± 0.02 µm/synapse; *n* = 61 synapses, four axons; pS129 [high]: 0.52 ± 0.02 µm/synapse; *n* = 67 synapses, four axons; one-way ANOVA *p* = 0.0004). However, in stark contrast to WT α-synuclein, treatment of synapses with excess pS129 did not change the total number nor the distribution of CCP/Vs at synapses (Figure 7, M and N) (Control: 3.16 ± 0.30 clathrin coats/synapse; *n* = 55 synapses, four axons; pS129 [low]: 2.83 ± 0.28 clathrin coats/synapse; *n* = 61 synapses, four axons; pS129 [high]: 2.93 ± 0.31 clathrin coats/synapse; *n* = 67 synapses, four axons; one-way ANOVA *p* = 0.8418). Indeed, three-dimensional synapse reconstructions revealed a normal number and distribution of CCP/Vs along the synaptic membrane (Figure 7, D–F, insets). Perhaps more surprisingly, the total membrane analysis revealed that pS129 induced a 31% loss of total synaptic membrane area at the high concentration, which we did not previously observe with WT α-synuclein (Figure 7O; Control: 1.62 ± 0.07 µm^2^/synapse; *n* = 55 synapses, four axons; pS129 [low]: 1.35 ± 0.06 µm^2^/synapse; *n* = 61 synapses, four axons; pS129 [high]: 1.11 ± 0.06 µm^2^/synapse; *n* = 67 synapses, four axons; one-way ANOVA *p* < 0.0001) (Busch *et al.*, 2014; Medeiros *et al.*, 2017, 2018; Banks *et al.*, 2020; Soll *et al.*, 2020). Thus, pS129 impaired SV trafficking with a distinct and severe, activity-dependent loss of SVs, which was only partially compensated by endocytic intermediates. The dramatic loss of SVs induced by excess pS129 at stimulated synapses, combined with the loss of total membrane within the immediate synaptic vicinity, suggested to us that pS129 was having additional impacts on activity-dependent SV clustering dynamics, as was suggested by the in vitro assays (see Figure 4).

### Excess pS129 α-synuclein induces activity-dependent SV dispersion in vivo

**FIGURE 8:**
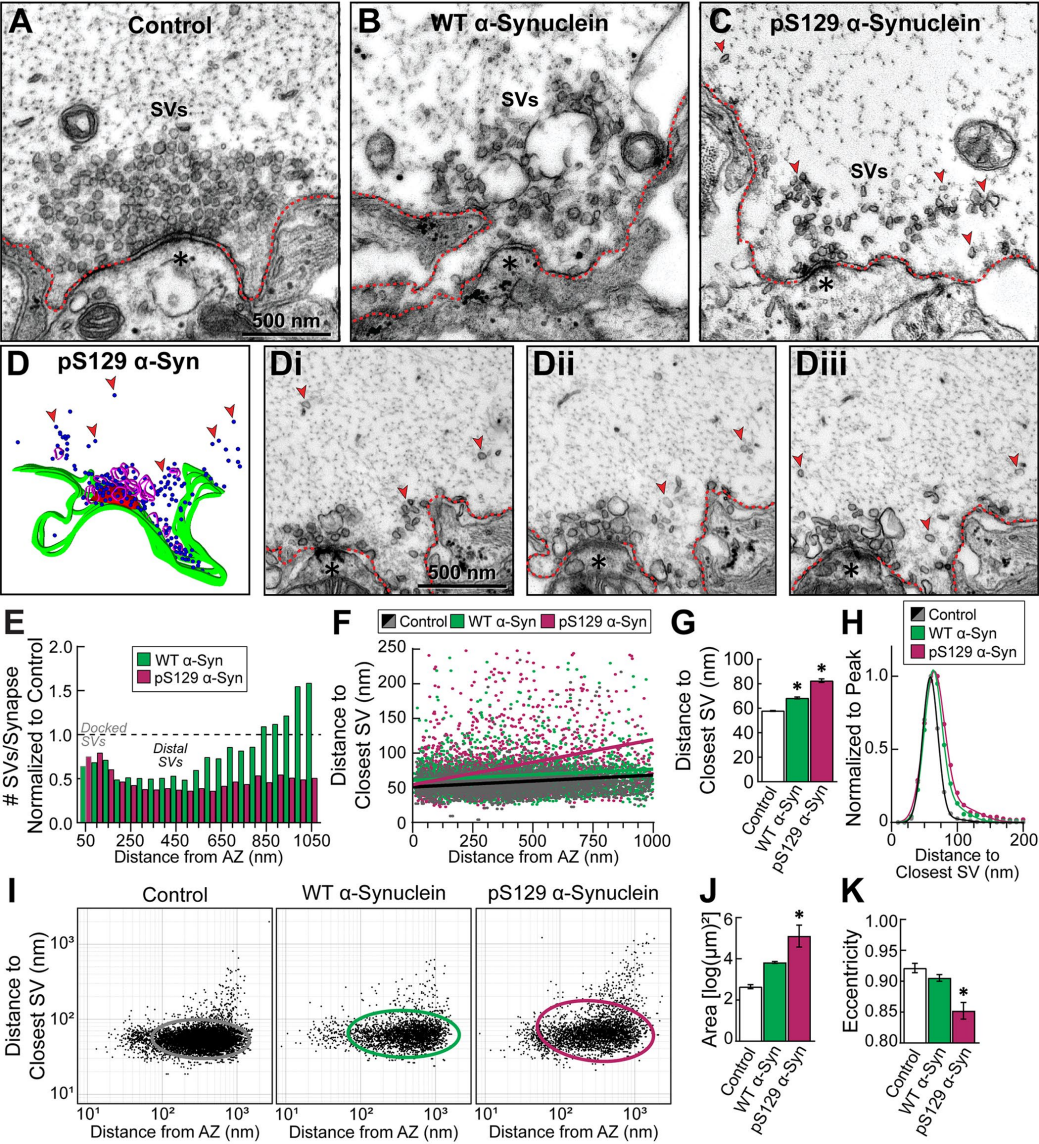
Excess pS129 α-synuclein induces SV declustering and dispersion at lamprey synapses. (A–C) EM micrographs showing a control, stimulated synapse (20 Hz, 5 min), compared with those treated with either WT or pS129 α-synuclein (“high” concentration: 10–20 μM). While WT α-synuclein induced moderate SV declustering, this was dramatically enhanced in the presence of excess pS129 α-synuclein, resulting in much smaller SV clusters. (D) three-dimensional reconstruction and (Di–iii) serial micrographs of a pS129 α-synuclein-treated synapse showing the SV dispersion. Red arrows indicate small, SV microclusters or single SVs dispersed away from the synapse. (E) SV distribution analysis. pS129 α-synuclein caused a reduction in SVs at all distances measured, which was greater than that caused by WT α-synuclein at distances >150 nm from the AZ. (F–K) By all measures, the nearest neighbor analyses revealed that pS129 α-synuclein caused greater dispersion of SVs, compared with WT α-synuclein. Note that the average distances between SVs significantly increased with pS129, compared with WT and controls (F–G). Furthermore, the nearest neighbor SV distributions fit a two summed Gaussian curve, and compared with controls, WT and pS129 α-synuclein caused a pronounced tail (H). A log–log density plot of data points from (F) also appears more disperse, leading to an increase in the area and eccentricity of the elliptical fits (H–K). Data in (F–K) are from *n* = 3,016–12,143 SVs (55–67 synapses, two to four axons/condition). * indicates *p* ≤ 0.05, compared with control, by one-way ANOVA.

We further examined the distribution of SVs at synapses treated with excess WT or pS129 α-synuclein (Figure 8). Compared to stimulated control synapses, both WT and pS129 α-synuclein (“high” concentration: 10–20 μM) induced a depletion of SVs near the active zone and dispersed the remaining SVs, causing a fragmentation of the SV cluster (Figure 8, A–C). However, there were clear differences in the SV clustering phenotypes. For WT α-synuclein, the remaining SVs remained clustered at the synapse and near the active zone (Figure 8B), commensurate with the local SV trafficking and endocytosis deficits that we previously reported (Busch *et al.*, 2014; Medeiros *et al.*, 2017, 2018; Banks *et al.*, 2020; Soll *et al.*, 2020). In contrast, with pS129 α-synuclein, there appeared to be a greater depletion and dispersion of SVs (Figure 8C; red arrows). Small bundles of SVs were often observed at distances away from the active zone, suggesting that pS129 crosslinks SVs into so-called “microclusters” in vivo, and individual SVs were also observed distally to synapses (Figure 8D, arrows). Compared to control synapses, the SV distributions for both WT and pS129 α-synuclein showed a similar ∼40–60% reduction at distances 0–500 nm from the AZ, including docked SVs (Figure 8E). However, while the SV distribution with WT α-synuclein gradually returned to and overshot the baseline, pS129 induced a sustained loss of ∼50% of SVs throughout the SV cluster, indicating a greater depletion of SVs (Figure 8E). A nearest-neighbor analysis of SVs was performed. At control and WT α-synuclein–treated synapses, nearest-neighbor distances between SVs increased with distance from the AZ, a positive correlation that was significantly more pronounced for pS129 α-synuclein (Figure 8F; Control: slope = 0.017 ± 0.001, R^2^ = 0.0227, *n* = 11,825 SVs [86 synapses, six axons]; WT: slope = 0.016 ± 0.003, R^2^ = 0.0111, *n* = 3321 SVs [30 synapses, two axons]; pS129: slope = 0.064 ± 0.004, R^2^ = 0.0654, *n* = 3321 SVs [67 synapses, four axons]; linear regression; one-way ANOVA *p* < 0.0001). In the presence of excess WT α-synuclein, SVs were on average ∼10 nm farther apart than at control synapses, a distance that increased to ∼25 nm with pS129 α-synuclein (Figure 8G; Control: x = 58.12 ± 0.30 nm, *n* = 12,143 SVs, *n* = 86 synapses, six axons; WT: x = 68.47 ± 0.73 nm, *n* = 3016 SVs, *n* = 30 synapses, two axons; pS129: x = 82.60 ± 1.46 nm, *n* = 3509 SVs, *n* = 67 synapses, four axons; one-way ANOVA *p* < 0.0001). Further analysis revealed that the distribution of nearest neighbor distances at control synapses was best fit by a two summed Gaussian (Control: R^2^ = 0.9992); however, WT and pS129 α-synuclein induced a progressive rightward shift of the peaks and the tails in these curves especially at distances >80 nm, reflective of SV dispersion (Figure 8H; WT: R^2^ = 0.9989; pS129: R^2^ = 0.9971). The nearest neighbor data from Figure 4F were converted into a log–log plot and fit with an ellipse, which further corroborated the SV dispersion caused by pS129 α-synuclein (Figure 8, I–K; Area - Control: 2.65 ± 0.10 log[µm]^2^, four axons; WT: 3.83 ± 0.05 log[µm]^2^, two axons; pS129: 5.11 ± 0.53 log[µm]^2^, four axons; one-way ANOVA *p* = 0.0006; Eccentricity - Control: 0.921 ± 0.007, four axons; WT: 0.905 ± 0.005, two axons; pS129: 0.85 ± 0.01, four axons; one-way ANOVA *p* = 0.0016). In contrast, at unstimulated synapses, introduction of excess pS129 did not cause SV dispersion by any of these measures (Supplemental Figure S4). Thus, only at stimulated synapses does pS129 α-synuclein induce severe SV declustering and dispersion away from the synaptic area, indicating an activity-dependent effect on SV clustering dynamics in vivo.

**FIGURE 9:**
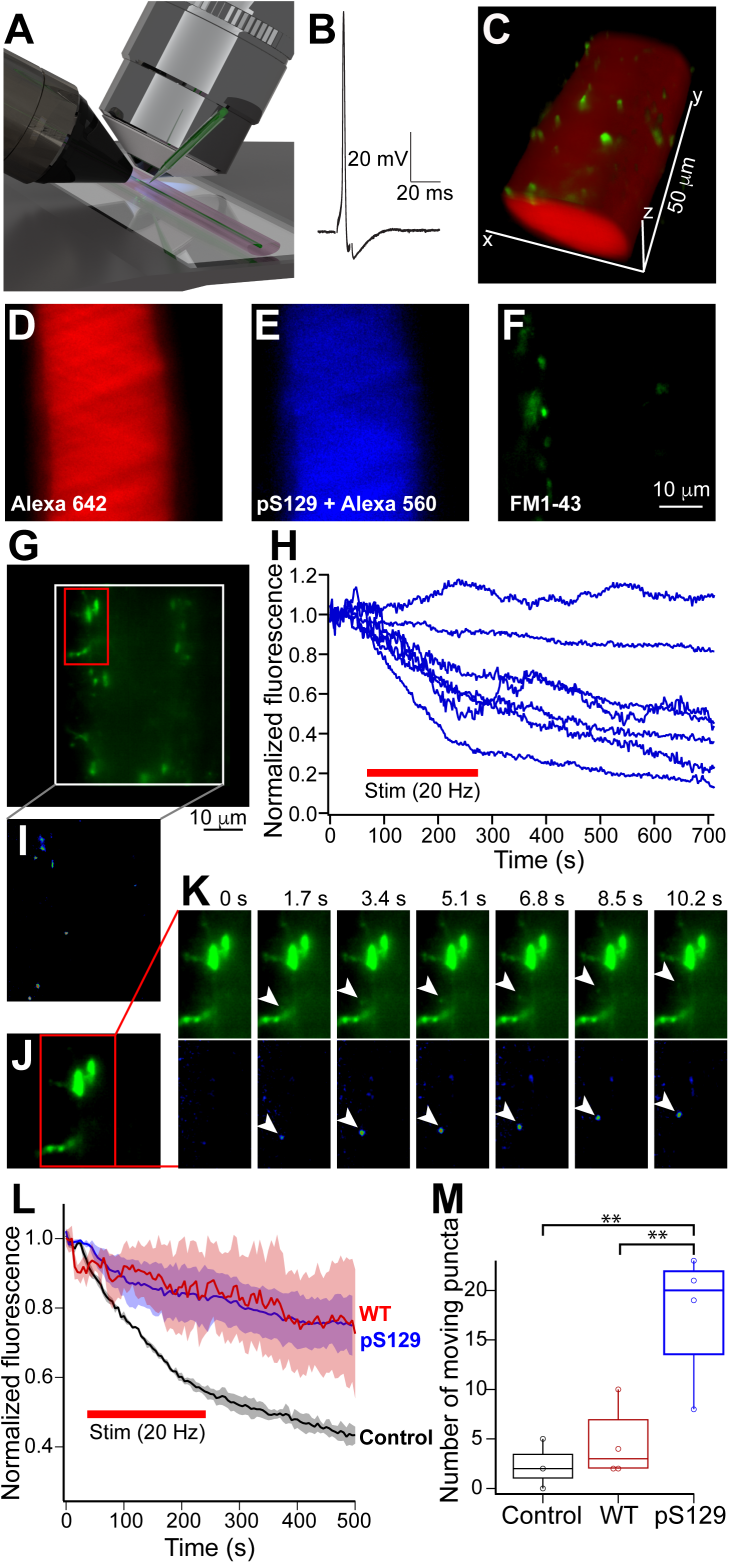
Live imaging confirms that pS129 α-synuclein disrupts SV trafficking and clustering in vivo. (A) Lamprey giant axons were imaged using lattice light sheet microscopy (LLSM) during physiological recordings and microinjections. (B) Action potentials were evoked through the recording microelectrode. (C) Giant axon filled with Alexa dye (red). Synapses were labeled with FM 1-43 dye (green) via activity-dependent uptake. (D–F) Axon prelabeled with Alexa 642 hydrazide and FM 1-43, followed by pS129 α-synuclein (“high” concentration: 10–20 μM) coinjected with Alexa 568 hydrazide. Images show a single LLSM plane through the axon. (G) Another axon with FM 1-43 labeled synapses and injected with pS129. Image shows a 5 µm thick z-stack. (H) Action potentials were applied at 20 Hz (4000 stimuli), which caused highly variable FM destaining in the presence of pS129. Each trace represents FM destaining measured from a single SV cluster. (I–K) During stimulation FM 1-43 labeled SV microclusters (resolution limited points [Potcoava *et al.*, 2021]) were mobile throughout the interior of the axon. Panel I show all mobile puncta in one time point z-stack, which was determined by expressing the frame as a ratio of the mean of 10 frames immediately prior. Panels J and K show an example of one of these SV microclusters leaving a larger synapse. (L) Average destaining kinetics reveal that WT and pS129 α-synuclein reduced the mean rate of destaining, as compared with control axons. Red bar indicates the stimulus (20 Hz, 4000 stimuli). Shaded areas show SEM. (M) pS129 caused a significant increase in the number of mobile FM1-43 labeled puncta within a 50-µm length of axon over a period of 200 s. ** indicates *p* < 0.01 by one-way ANOVA. Data in (L and M) are from *n* = 22-33 synapses, three to four axons per experimental condition.

To visualize the SV dynamics in living axons, we labeled synapses with FM 1-43, a lipophilic dye that fluoresces upon intercalation into the PM outer leaflet and internalization via SV endocytosis (Betz and Bewick, 1992). FM dyes have been used previously to label lamprey giant RS synapses and monitor exocytosis/endocytosis kinetics (Kay *et al.*, 1999; Photowala *et al.*, 2006; Bleckert *et al.*, 2012). Here, we adapted this technique to a customized lattice light sheet microscope that enables very rapid volume reconstruction (Figure 9A) (Chen *et al.*, 2014; Potcoava *et al.*, 2021). Axons were preinjected with a long wavelength dye (Alexa 642 hydrazide) and then stimulated at 5 Hz (2000 stimuli) to evoke action potentials (Figure 9B) while superfusing with FM1-43, to label the recycling SVs, followed by clearing with Advasep-7 (Figure 9C) (Kay *et al.*, 1999). Next, FM labeled axons were either left untreated or coinjected with WT or pS129 α-synuclein (“high” concentration: 10–20 μM) and a spectrally distinct marker (Alexa 568) to confirm successful protein injection into the same axon (Figure 9, D–G). Subsequent stimulation at 20 Hz caused normal destaining of the FM-labeled control axons, but an unusually high, variable rate of destaining of the FM-labeled SV clusters in the axons injected with WT or pS129 α-synuclein (Figure 9, H and L). At individual synapses, the destaining kinetics ranged from a lack of destaining to atypically rapid destaining (Figure 9H). When averaged from a larger population of synapses, the destaining kinetics with WT or pS129 α-synuclein were slower than in controls, but did not reach significance, while the increased variance of the decay rate was significant, indicating complex impacts on SV trafficking (Figure 9L; Control: τ = 213 ± 4 s, *n* = 24 synapses, three axons; WT: τ = 5056 ± 3383 s, *n* = 22 synapses, four axons; pS129: τ = 8403 ± 7053 s, *n* = 33 synapses, four axons; one-way ANOVA, *p* = 0.1; F[3] = 19, *p* = 4 × 10^–7^). Closer examination of SV clusters, which was enabled by the lattice light sheet, revealed diffraction limited FM1-43 labeled particles moving within the axon after injection of pS129 (Figure 9, I–K; Supplemental Movie 1). An example of a particle leaving an SV cluster is shown (Figure 9, J and K). Speed of movement was variable up to a maximum of 1.2 µm/s. Such particles were not evident in 20 Hz stimulated control axons that were not injected with pS129 α-synuclein (Supplemental Movie 2). Indeed, quantification revealed very small numbers of these mobile puncta in untreated control axons or those injected with WT α-synuclein, while nearly eightfold more mobile puncta were visible in the pS129 injected axons (Figure 9M; Control: 2.3 ± 1.5 puncta, *n* = 24 synapses, three axons; WT: 4.5 ± 2.2 puncta, *n* = 22 synapses, four axons; pS129: 17.8 ± 3.9 puncta, *n* = 33 synapses, four axons; one-way ANOVA *p* = 0.006). These data provide further functional evidence that SV cycling is impaired and that SVs dynamically disperse in microclusters in the presence of excess pS129 α-synuclein at stimulated synapses.

**Figure:** Movie S1 Movie showing FM 1‐43 labeled SVs during 20 Hz stimulation, in the presence of pS129. Note the moving particles, which are ‘microclusters’ of SV.

**Figure:** Movie S2 Movie showing FM 1‐43 labeled SVs during 20 Hz stimulation, without pS129. The moving particles are all but absent.

## DISCUSSION

**FIGURE 10:**
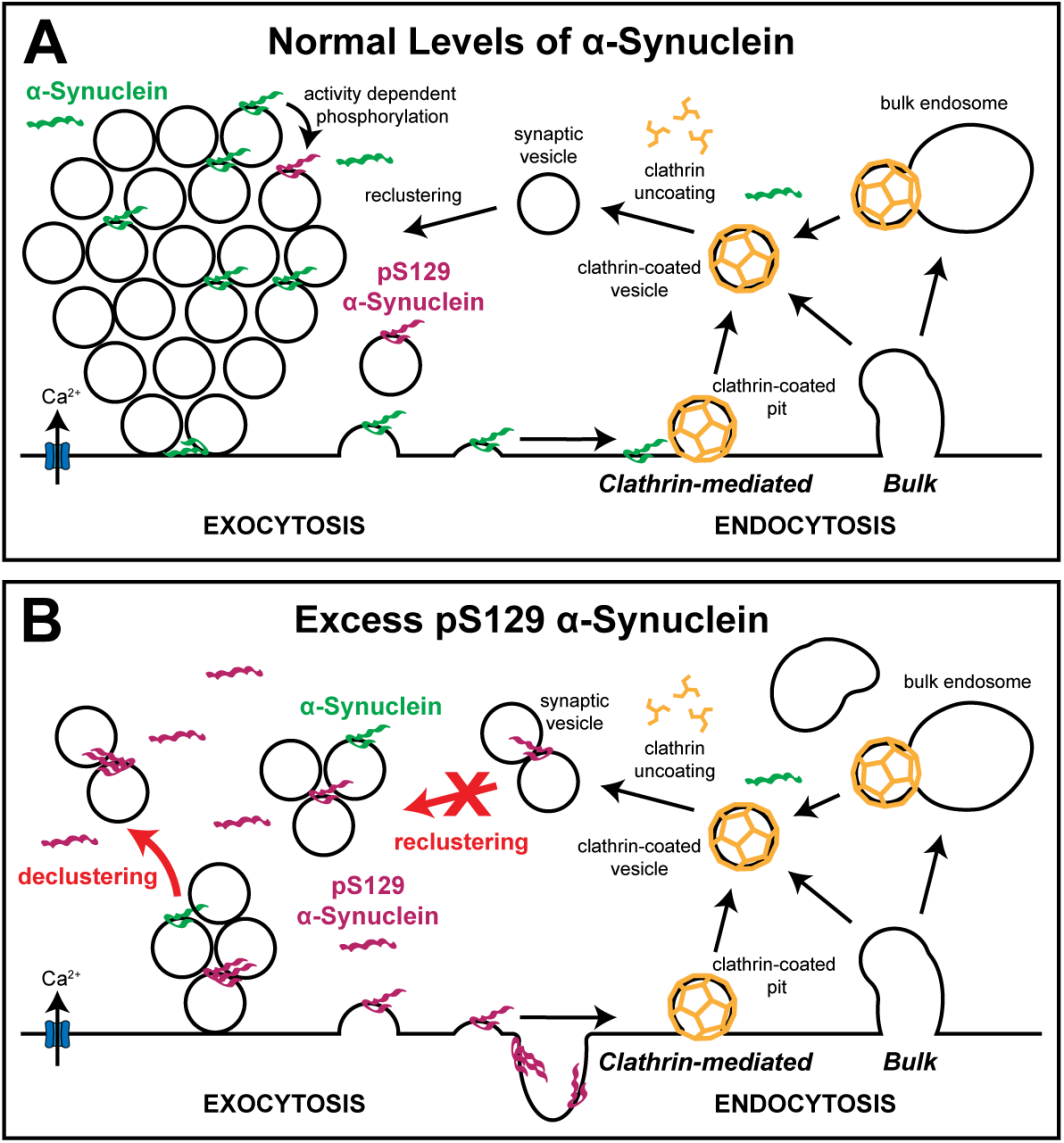
Working model illustrating how pS129 α-synuclein impacts synapses. (A) Diagram showing normal SV trafficking under physiological conditions. α-Synuclein participates in SV exocytosis, endocytosis, and SV clustering. Upon activity, some α-synuclein is pS129, which regulates synaptic transmission and plasticity (Ramalingam *et al.*, 2023). Lamprey synapses are known to exhibit clathrin-mediated and bulk endocytosis for recycling SVs, though other nonclathrin mechanisms may also participate, which are not shown for simplicity. (B) When introduced acutely to synapses, excess pS129 α-synuclein causes a severe loss of SVs, which is likely due to aberrant clustering/declustering and/or altered postendocytic reclustering. SVs appear to break into small microclusters of SVs, which may be facilitated by the enhanced membrane binding and oligomerization of pS129. Such alterations of the SV clustering dynamics would allow SVs to float away from the synapse exacerbating synaptic transmission defects.

By taking advantage of the unique features of lamprey giant RS synapses, we have now demonstrated that acute introduction of excess pS129 α-synuclein, mimicking increased levels observed in PD and DLB, induced rapid, activity-dependent impacts on SV clustering and vesicle trafficking at synapses. As a consequence, SVs became declustered, leading to a severe depletion of SVs and loss of total membrane from the synaptic area (Figure 10). Supporting this conclusion, the impacts of the pS129 modification on SV clustering were observed via multiple independent methods, including the in vitro turbidity assays (Figure 4), ultrastructural analyses (Figures 7 and 8), and functional imaging (Figure 9). Although the vesicle trafficking defects caused by excess pS129 α-synuclein may appear to be consistent with an inhibition of SV endocytosis, given the loss of SVs and expanded PM (Figure 7), as well as the altered FM 1-43 destaining kinetics (Figure 9), several lines of evidence suggest that impaired endocytosis may not be the dominant effect. First, we also observed an expansion of the PM at unstimulated synapses treated with pS129 α-synuclein (Figure 6), which is likely reflective of its avid membrane binding capacity. Second, excess pS129 did not induce any obvious impacts on clathrin-mediated SV recycling, unlike previous results obtained with monomeric and dimeric WT α-synuclein (Busch *et al.*, 2014; Medeiros *et al.*, 2017; Banks *et al.*, 2020; Soll *et al.*, 2020). This is quite interesting because α-synuclein has been proposed to play a physiological role in regulating CME through AP2- and PI(4,5)P_2_-mediated events (Ben Gedalya *et al.*, 2009; Vargas *et al.*, 2014, 2021; Schechter *et al.*, 2020). Third, the complex changes in the FM 1-43 destaining kinetics induced by pS129, coupled with the appearance of mobile particles, was unique and did not follow the typical pattern for a purely exo/endo-phenotype, though this certainly warrants further investigation (Cheung *et al.*, 2010; Scott *et al.*, 2010; Scott and Roy, 2012). Finally, in contrast to pS129 α-synuclein, excess WT α-synuclein produced more of a clear endocytic phenotype, where the loss of SVs was completely compensated by an increase in PM extensions and endocytic intermediates, indicating a local redistribution of the membranes at synapses (Busch *et al.*, 2014; Medeiros *et al.*, 2017; Banks *et al.*, 2020). Although there were no obvious impacts of excess pS129 on CME, we acknowledge the possibility that it may alternatively affect clathrin-independent pathways for endocytosis (Chanaday *et al.*, 2019), which has not yet been directly tested. Supporting an effect on endosomal trafficking, both WT α-synuclein, pS129 α-synuclein, and other variants tested all cause an increase in the number and/or size of large endosome-like vesicles (Busch *et al.*, 2014; Medeiros *et al.*, 2017; Soll *et al.*, 2020; Roman-Vendrell *et al.*, 2021), which is also consistent with α-synuclein’s known interactions with several Rab GTPases (Chen *et al.*, 2013; Breda *et al.*, 2015).

Our data would therefore suggest that the pS129 modification may serve as a “switch” to shift α-synuclein away from CME toward some other function such as regulation of SV clustering/declustering dynamics. Although acute introduction of WT α-synuclein did induce mild SV declustering at lamprey synapses, it was much less severe than what was observed with pS129 (Figures 8 and 9) (Banks *et al.*, 2020). Thus, pS129 α-synuclein impairs SV trafficking and SV clustering/reclustering in a manner that is distinct from WT α-synuclein, further contributing to the growing body of evidence that different molecular species of α-synuclein induce distinct impacts at synapses (Busch *et al.*, 2014; Medeiros *et al.*, 2017; Banks *et al.*, 2020; Soll *et al.*, 2020; Roman-Vendrell *et al.*, 2021). Moreover, these data further reveal that single PTMs, such as pS129, can substantially alter α-synuclein’s biochemical properties (Figures 3 and 4) and physiological impacts (Figures 7–9) (Paleologou *et al.*, 2008; Parra-Rivas *et al.*, 2022; Ramalingam *et al.*, 2023). Given that excess pS129 α-synuclein caused a greater depletion of SVs and loss of total synaptic membrane at the lamprey synapses than its WT counterpart, this would also suggest that its pathological accumulation at synapses may ultimately be more deleterious to SV trafficking and neurotransmission, though this will need to be explored further.

The physiological and pathological impacts of α-synuclein and its variants on synapses and in cells are highly correlated with their membrane binding properties (Busch *et al.*, 2014; Burre *et al.*, 2015; Wrasidlo *et al.*, 2016; Dettmer *et al.*, 2017). Previous studies revealed that the pS129 modification does not substantially alter the micelle-bound alpha helical structure of α-synuclein, consistent with the intact N-terminal domain (Figure 1A) (Paleologou *et al.*, 2008). We further demonstrate here that this PTM does not alter the overall lipid binding profiles of α-synuclein, as both WT and pS129 selectively bind to anionic lipids that are generally enriched in SVs, membranes, and mitochondria (Figure 2). However, the pS129 modification did enhance binding to purified synaptic membranes (Figure 3) and α-synuclein mediated SV clustering in vitro (Figure 4), consistent with an important role for the C-terminus in modulating membrane and vesicle interactions (Lautenschlager *et al.*, 2018). Although a priori it may seem counterintuitive that pS129 binds even better to synaptic membranes and enhances SV clustering, given the addition of negative charge, there are several plausible explanations. One possibility is that the increased oligomerization of pS129 may help to crosslink SVs in such a way that strengthens and stabilizes membrane binding and promotes tighter SV microclustering. Alternatively, or in addition, the conformational changes induced by phosphorylation of α-synuclein may help to promote stronger binding to its synaptic interaction partners. Indeed, phosphorylation of serine 129 reduces long range intramolecular interactions within α-synuclein, leading to more extended conformations of the protein (Paleologou *et al.*, 2008). Perhaps this is why phosphorylation of α-synuclein at S129 enables stronger interactions with its major synaptic binding partners, VAMP2 and synapsin (Parra-Rivas *et al.*, 2022). Either of these mechanisms would have the net effect of enhancing or stabilizing α-synuclein binding to SVs. Therefore, our working model is that pS129 α-synuclein stabilizes small SV microclusters, leading to a dysregulation of SV clustering and reclustering during vesicle recycling and allowing the vesicles to escape away from the synaptic area (Figure 10). The speed of movement of the FM-labeled SV puncta is consistent with fast axonal transport (Supplemental Movie 1), and going forward it will be important to uncover the transport mechanisms.

Another important aspect to consider is the activity-dependence of the synaptic phenotypes caused by excess α-synuclein. Both WT α-synuclein (Busch *et al.*, 2014) and pS129 α-synuclein (Figures 6–9) impair SV trafficking during high frequency stimulation (20 Hz), with little or no effect in the absence of stimulation. The mechanisms underlying this phenomenon are unknown, but likely involve the stimulation-dependent increase in presynaptic calcium and the consequent calcium-induced enhancement of α-synuclein binding to synaptic membranes. A recent study reported that six to eight calcium ions bind to the C-terminus of WT α-synuclein, thereby enhancing its interaction with SVs by neutralizing negative charges on C-terminal residues (Lautenschlager *et al.*, 2018). Calcium also enhances α-synuclein-mediated SV “microclustering” in vitro (Aryal *et al.*, 2021). Thus, the C-terminal calcium binding might provide a mechanism by which α-synuclein can “sense” and respond to neuronal activity. That said, the previous studies only tested WT α-synuclein, leaving open the possibility that the calcium-binding properties may differ for pS129 α-synuclein because the amino acid residues responsible for calcium binding and enhanced SV interaction overlap with serine 129 (Lautenschlager *et al.*, 2018). The current working model is that the stimulation-dependent rise in intracellular calcium triggers both generation of pS129 through calcineurin-dependent activation of PLK2 (Ramalingam *et al.*, 2023) and stabilization of α-synuclein on SVs (Lautenschlager *et al.*, 2018; Aryal *et al.*, 2021). The enhanced membrane binding and oligomerization of pS129 apparently leads to tighter “microclustering” of SVs during activity-dependent SV trafficking, which causes a more dominant impairment of SV clustering/reclustering dynamics and leads to SV declustering and dispersion from the synapse (Figure 10). Additional tests of the model are needed, including a better understanding of how pS129 α-synuclein responds to calcium, as well as the mechanisms by which α-synuclein and synapsin I cooperate throughout the different stages of the SV cycle.

We estimate the pS129 α-synuclein concentration delivered to lamprey synapses to be ∼5–20 μM, a range which is in line with our estimates of its pathological accumulation in disease states. Measurements of physiological α-synuclein at synapses range from ∼3–6 μM (Westphal and Chandra, 2013) to ∼20 μM (Wilhelm *et al.*, 2014). If 4% of total α-synuclein is phosphorylated at serine 129 (Fujiwara *et al.*, 2002), this would suggest that pS129 α-synuclein concentration at synapses is normally ∼0.1–0.8 μM under physiological conditions. pS129 α-synuclein is reported to increase 30-fold in DLB brains (Anderson *et al.*, 2006) with 20–25% of that being at synapses (Colom-Cadena *et al.*, 2017), suggesting that pS129 goes up to ∼5 μM at synapses in DLB brains and even higher (up to 2.5-fold more) upon neuronal activity (Parra-Rivas *et al.*, 2022; Ramalingam *et al.*, 2023). Thus, our experimental conditions roughly match the estimated concentrations of pS129 α-synuclein at synapses in diseased brains. However, we acknowledge that we do not know the aggregation status of the protein after injection, although extensive aggregation is highly unlikely due to the acute nature of these perturbations that occur in less than 1 hour. Recent evidence from both zebrafish and mouse models suggests that pS129 accumulation at synapses and α-synuclein aggregation are separable events (Weston *et al.*, 2021a, 2021b). We therefore propose that in diseased brains, pS129 α-synuclein generated by neuronal activity during bouts of high frequency stimulation can rapidly bind and oligomerize on synaptic membranes, and if not compensated somehow, this can lead to SV clustering/reclustering and trafficking deficits (Figure 10). Further oligomerization, aggregation, or strain conformations of pS129 α-synuclein that increase over time would add additional complexity to its synaptic impacts in PD, DLB, and other synucleinopathies.

At present, it is unclear whether the accumulation of pS129 α-synuclein in the pathological state is due to increased phosphorylation of α-synuclein by PLK2, decreased dephosphorylation by PP2A phosphatase, an attempt by neurons to increase neuronal activity in the face of degenerative processes, or some other mechanism that causes an imbalance in the homeostasis of this PTM (Ramalingam *et al.*, 2023). Interestingly, PLK2 has been reported to be upregulated by two- to threefold in the brains of AD and DLB patients (Mbefo *et al.*, 2010). In addition, PP2A is dysregulated in PD and DLB brains (Park *et al.*, 2016). Moreover, several PTPA variants that impair activation of PP2A were recently reported to be linked to early onset parkinsonism with intellectual disability (Fevga *et al.*, 2023). It is unknown whether these changes in PLK2 and PP2A expression levels or activity are causal drivers of the disease pathologies or whether they are compensatory responses to deal with elevated pS129 α-synuclein. Regardless, targeting PLK2 or PP2A activity as a means to titrate pS129 and WT α-synuclein back to physiological levels may be emerging as a viable strategy that has potential for therapeutic value in PD and DLB.

Besides pS129, there are a number of other α-synuclein PTMs that are worth further exploration, particularly because some appear to have neuroprotective effects (Zhang *et al.*, 2019). For example, pS87 α-synuclein, which is elevated in DLB brains, exhibits reduced alpha helicity upon membrane binding, inhibits α-synuclein fibrillation, and therefore reduces neurotoxicity in rodent models of PD (Paleologou *et al.*, 2010; Oueslati *et al.*, 2012). In addition, O-GlcNAcylation of α-synuclein can radically affect its biochemical activity in fibrillation assays with some glycosylated variants showing neuroprotection in rodent models of PD (Marotta *et al.*, 2015; Lewis *et al.*, 2017; Levine *et al.*, 2019; Balana *et al.*, 2023). O-GlcNAcase inhibitors are currently in clinical trials for tauopathies (Selnick *et al.*, 2019), and therefore it will be important to know whether these drugs might affect α-synuclein functions and dysfunctions at synapses in order to ward off any undesirable effects. Going forward, it will be essential to fully understand the impacts of these and other α-synuclein PTMs in both the physiological and pathological states, as they are likely to have important roles in both normal brain function and in disease.

## MATERIALS AND METHODS

Request a protocol through *Bio-protocol*.

### SDS–PAGE and Western blotting

Recombinant WT human α-synuclein (MW 14,460 Da) was expressed in *E. coli* and purified to >95% purity by rPeptide (Watkinsville, GA). pS129 α-synuclein (MW 14,540 Da) was generated by chemical ligation of a recombinant α-synuclein fragment (a.a. 1-84) and a synthetic phospho-peptide (a.a. 85-140), and further purified to >95% purity by HPLC (Proteos, Kalamazoo, MI). Equal amounts of pS129 and WT α-synuclein were run on a 12% SDS–PAGE gel (2 μg for Coomassie; 250 or 500 ng for Western blots). In some experiments, we instead ran total protein lysates prepared from rat brains (20 μg) or lamprey central nervous system (CNS; 40 μg) to test antibody cross-reactivity against endogenous synucleins in these species. The proteins were then transferred to nitrocellulose membrane and subsequently detected with α-synuclein antibodies. A rabbit polyclonal anti-pan-synuclein antibody (1:1000; ab53726; Abcam; Waltham, MA) was used to detect total α-synuclein. Phosphoserine129 modification was independently confirmed with three pS129-specific α-synuclein antibodies: a mouse monoclonal (1:1000; 825701 BioLegend; San Diego, CA); a rabbit polyclonal (1:1000; SPC-742; StressMarq; Victoria, British Columbia); and a rabbit monoclonal (1:1000; D1R1R; Cell Signaling Technology; Danvers, MA). Secondary antibodies included HRP-conjugated goat antirabbit or goat antimouse IgG (H+L), as appropriate (1:1000; Thermo Fisher Scientific; Waltham, MA). Protein bands were detected using ECL; Pierce ECL Western Blotting Substrate; Thermo Fisher Scientific; Waltham, MA) and imaged using an Azure Imaging System 300 (Azure Biosystems; Dublin, CA).

### Protein-lipid overlay assays

Protein-lipid overlay assays were performed using Membrane Lipid Strips^TM^ (Echelon Biosciences; Salt Lake City, UT). After blocking, (5% dry milk in 20 mM phosphate buffer, pH 7.4, 0.05% Tween-20), Membrane Lipid Strips^TM^ were first incubated with 10 μg/ml WT or pS129 α-synuclein for 1 h at room temperature (RT). Membrane Lipid Strips^TM^ were then washed for 2 min in 1% dry milk in 20 mM phosphate buffer, pH 7.4, 0.05% Tween-20. Following the wash, the strips were incubated in primary antibodies for 1 h 15 min at RT followed by two 15-min washes at RT. The primary antibody used to recognize WT α-synuclein was a mouse monoclonal antibody specific for human α-synuclein (Abcam; ab1903 [clone 4D6]; 1:1000). pS129 α-synuclein was recognized using a rabbit monoclonal antibody (Cell Signaling Technology; D1R1R; 1:1000). Finally, the Membrane Lipid Strips^TM^ were incubated for 1 h 15 min in secondary anti-bodies, which included HRP-conjugated goat anti-mouse or goat anti-rabbit IgGs (H+L), respectively (Thermo Fisher Scientific; 1:1000). After washing, any membrane bound WT or pS129 α-synuclein was detected using ECL and imaged on the Azure Imaging System 300. α-Synuclein binding was subsequently quantified in FIJI by measuring the background-subtracted intensity of the ECL signal associated with each lipid on the Membrane Lipid Strips^TM^ from *n* = 4–7 independent experiments. Graphs were generated and statistical analyses performed in Prism 9.5 (GraphPad; Boston, MA).

### Membrane binding assays

Purified synaptosomes stripped of associated proteins, and cytosol preparations, were made from *n* = 8 and *n* = 2 mouse brains, respectively, as previously described (Soll *et al.*, 2020; Vargas *et al.*, 2021). For the membrane binding assays, 200 μg synaptic membranes and 250 μg cytosolic proteins were mixed in cytosolic buffer (25 mM HEPES-NaOH, pH 7.4; 120 mM potassium glutamate; 2.5 mM magnesium acetate; 20 mM KCl; and 5 mM EGTA-NaOH, pH 8.0) to a final volume of 500 μl, and supplemented with 2 μM recombinant WT or pS129 α-synuclein. Mixtures were incubated for 15 min at 37°C, after which samples were ultracentrifuged at 100,000 × *g* for 30 min at 4°C. The pellets, which contained the synaptic membranes with bound proteins, were resuspended in 500 μl cytosolic buffer, centrifuged at 4°C, and resuspended in a final volume of 90 μl cytosolic buffer. For each sample, 20 μl aliquots were run on a 12% SDS–PAGE gel, followed by Western blotting against α-synuclein using a pan-synuclein antibody (1:1000; ab53726; Abcam). Western blotting against N-cadherin using a rabbit polyclonal antibody (1:1000; GTX127345; GeneTex) served as a loading control for the membranes. Protein bands were detected using ECL, quantified using FIJI, and then normalized to the membrane marker N-cadherin to control for any loading variability. All graphs were made and statistical analyses performed in GraphPad Prism 9.5.

### In vitro vesicle clustering assays

#### Recombinant Proteins.

EGFP-tagged synapsin 1 (*Rattus norvegicus*; NCBI Reference Sequence: NM_019133.2) was expressed in Expi293F cells (Thermo Fisher Scientific) and purified, as previously described (Milovanovic *et al.*, 2018; Hoffmann *et al.*, 2021). Untagged recombinant human pS129 α-synuclein was obtained from Proteos WT human α-synuclein was expressed in *E.coli* (BL21) from a pET28 plasmid (Novagen; Darmstadt, Germany). Briefly, *E. coli* expressing untagged human α-synuclein were lysed in ice-cold phosphate-buffered saline (PBS) supplemented with protease inhibitors (Complete EDTA-free, Roche) using a high-pressure homogenizer and cleared via centrifugation (30 min, 50,000 × *g*). Nucleic acids were then precipitated by adding streptomycin sulfate (10 mg/ml) followed by constant agitation for 30 min at RT and then centrifugation (50,000 × *g* for 30 min). Proteins were precipitated from the soluble supernatant by slowly adding ammonium sulfate to a final concentration of 0.36 g/ml with constant agitation for 60 min at 4°C. The protein pellet (centrifugation: 30 min, 50,000 × *g*) was resuspended in 10 ml PBS and boiled for 20 min with stirring every few minutes. Insoluble proteins were pelleted by centrifugation (30 min at 50,000 × *g*, 4°C), and the WT α-synuclein containing supernatant was dialyzed against 15 mM Tris base solution overnight. Dialyzed α-synuclein was subjected to anion exchange chromatography (HiPrep Q FF 16/10, ÄKTA pure 25M; Cytiva Life Sciences; Marlborough, MA) in 25 mM Tris-HCl pH 7.7 (Buffer A). Washing was done with 150 mM NaCl, and α-synuclein was eluted with 250 mM NaCl (both in Buffer A). Elution fractions were concentrated and subjected to size exclusion chromatography (Superdex 75 Increase 10/300, ÄKTA pure 25M; GE Healthcare; Chicago, IL) in a buffer containing 25 mM Tris-HCl (pH 7.4), 150 mM NaCl, 0.5 mM TCEP. α-Synuclein concentration was determined at 280 nm using a molar extinction coefficient of 5960 M^–1^ cm^–1^. Proteins were snap-frozen and stored at –80 C° until use.

#### SV purification.

Native SVs were prepared from rat brains, as described in (Huttner *et al.*, 1983; Hoffmann *et al.*, 2021). Briefly, synaptosomes prepared from 20 rat brains were subjected to hypoosmotic shock. Released SVs were purified by centrifugation on a continuous sucrose density gradient and, in a final step, subjected to size-exclusion chromatography on controlled pore glass beads (300-nm diameter; Hoffmann *et al.*, 2021).

For the basic in vitro vesicle clustering assays, 6 µM of EGFP-synapsin 1 was mixed with 2 µM α-synuclein (WT or pS129) and 5 nM native SVs in a total volume of 30 µl (on ice), and transferred to a 384-well microtiter plate (#781906, Greiner Bio-One). To examine α-synuclein–mediated effects on SV clustering, 8 or 24 µM of α-synuclein (WT or pS129) was mixed with 5 nM native SVs in the absence of synapsin I. For all experiments, optical density (OD) measurements were taken at 405 nm every 10 min using a Synergy H1 Hybrid Multi-Mode Microplate Reader (BioTek Instruments; Charlotte, NC), as described in (Hoffmann *et al.*, 2021). All measurements were performed in 25 mM Tris-HCl (pH 7.4), 150 mM NaCl, 0.5 mM TCEP. Graphs were made using GraphPad Prism 9.5.

For visualization of α-synuclein induced SV microclusters, samples from the in vitro clustering assays were collected at 0- and 24-h incubation time points and prepared for electron microscopy as follows. Freshly glow-discharged, formvar/carbon-coated 300-mesh copper grids (EM Sciences) were floated on 5 µl droplets of each sample and incubated for 1 min. Grids were then washed six times on drops of ddH_2_O before being floated on a droplet of 1% uranyl acetate for 1 min. After two additional washes in ddH_2_O, excess liquid was wicked off with filter paper, and grids were air dried for >1 h. SVs were imaged at 37,000x magnification on a JEOL-200CX TEM.

### Axonal microinjections and stimulation

Microinjections of α-synuclein into lamprey giant RS axons were performed as previously described (Busch *et al.*, 2014; Banks *et al.*, 2020). All vertebrate animal procedures were approved by the Institutional Animal Care and Use Committee at the Marine Biological Laboratory in accordance with standards set by the National Institutes of Health. Briefly, late-stage larval lampreys (10–13 cm; M/F) were anesthetized in 0.1 g/L tricaine methanesulfonate (Syncaine; Syndel, Ferndale, WA). Next, 2-3 cm segments of spinal cords were dissected out and pinned ventral side up in a Sylgard 184-lined petri dish (Ellsworth Adhesives; Germantown, WI). Dissected spinal cords were submerged in fresh, oxygenated lamprey ringer solution (100 mM NaCl, 2.1 mM KCl, 1.8 mM MgCl_2_, 4 mM sucrose, 2 mM HEPES, 0.5 mM L-glutamine, 2.6 mM CaCl_2_, pH 7.4). WT and pS129 human α-synuclein were dialyzed into lamprey internal solution (180 mM KCl, 10 mM HEPES-K+, pH 7.4) to a concentration of 185–195 μM, after which they were microinjected directly into giant RS axons using small puffs of nitrogen (5–10 ms; 40 psi, 0.2 Hz) delivered via a Toohey Spritzer. α-Synuclein protein was coinjected with a fluorescent dye of similar molecular weight (0.1 mM FITC dextran, 10 kDa; Thermo Fisher Scientific) in order to estimate the final axonal concentration. After injection and diffusion in the axons, α-synuclein proteins were diluted to 1/10th or 1/20th of the starting pipet concentration, such that the final axonal concentration was approximately 10–20 μM. Injected axons were either fixed immediately without stimulation, or stimulated (20 Hz, 5 min) before fixation. For whole mount IF experiments, spinal cords were fixed in 4% paraformaldehyde in 0.1 M PBS, pH 7.4 for >3 h at RT and then overnight at 4°C. For electron microscopy experiments, the spinal cords were fixed in 3% glutaraldehyde, 2% paraformaldehyde in 0.1 M sodium cacodylate buffer, pH 7.4 for >3 h at RT and then overnight at 4°C.

### Whole-mount IF and image analysis

For whole-mount IF experiments, the injected, fixed spinal cords were first incubated for 1 h in 10% Normal Goat Serum blocking solution (Thermo Fisher Scientific) with 0.5% Triton X-100 followed by an overnight incubation at 4°C in primary antibodies overnight. Primary antibodies included: a mouse monoclonal anti-SV2 antibody (1:100) that was deposited to the Developmental Studies Hybridoma Bank by K. M. Buckley (Buckley and Kelly, 1985); a rabbit monoclonal human α-synuclein antibody (Abcam, ab138501 [MJFR1], 1:100); and a rabbit monoclonal anti-pS129 α-synuclein antibody (Cell Signaling [D1R1R]; 1:100). The SV2 antibody recognizes SV glycoprotein 2 within SVs in all vertebrates tested, including lampreys (Buckley and Kelly, 1985; Busch *et al.*, 2014; Banks *et al.*, 2020). After labeling with primary antibodies, spinal cords were washed for 5 × 1 h in wash buffer (20 mM Na phosphate buffer, 450 mM NaCl, and 0.3% Triton X-100; pH 7.4), followed by overnight secondary antibody incubations at 4°C. Secondary antibodies used were AlexaFluor^TM^ Plus 488 goat anti-rabbit IgG (H+L; Thermo Fisher Scientific) and AlexaFluor^TM^ Plus 594 goat anti-mouse IgG (H+L; Thermo Fisher Scientific), as appropriate. After washing 5 × 1 h in wash buffer, the spinal cord preps were mounted on glass slides with a 1.5-mm glass coverslip and sealed with ProLong Gold antifade reagent (Thermo Fisher Scientific).

The giant axons of interest were initially identified by the AlexaFluor^TM^ 488 signals associated with the injected pS129 or WT α-synuclein protein. Then, images of giant synapses within these axons were acquired using a 63x Plan-Apochromat objective (1.4 NA, 2 × optical zoom) on a Zeiss LSM980 Axio Examiner microscope with Airyscan2. Confocal images were subsequently processed using the Airyscan processing method on Zeiss Zen 3.4 (Blue edition, Carl Zeiss Microscopy GmbH). Processing strength was within ±1 unit from the “auto” strength number for all images: 488 channels (α-synuclein) = 6.9 and 594 channel (SV2) = 6.4.

To determine the overlap between injected α-synuclein and SV2, a histogram intensity analysis was conducted on giant RS synapses (*n* = 29 and 30 synapses; four and five axons; three animals) where a straight-line ROI was drawn perpendicular to the axon through a synapse to acquire the fluorescence intensity profiles for both SV2 and α-synuclein. The peak of the SV2 channel (red) was set as the center distance for each channel, all fluorescence profiles were then averaged and normalized to their respective peaks. The peak of the α-synuclein channel was then calculated relative to SV2. Fluorescent profiles were drawn using FIJI, and data were graphed in Prism 9.5.1 (GraphPad Software, LLC.).

To determine the colocalization between SV2 and injected α-synuclein, we analyzed the overlap of fluorescence associated with each channel using a MCC (Manders *et al.*, 1993). ROIs were selected that had an area of 60–75 µm^2^ and *n* = 2–9 synapses. An automatic thresholding algorithm was used to determine the background (Costes *et al.*, 2004), which was achieved by iteratively dropping the threshold for the SV2 channel until the Pearson’s Correlation Coefficient (PCC) crossed zero (Manders *et al.*, 1993). The α-synuclein channel threshold is related to the SV2 threshold through a linear least-squares fit of the α-synuclein versus SV2 intensity data (Costes *et al.*, 2004). MCC values were calculated using code written in MATLAB and verified in the FIJI plugin JACoP. To determine the amount of α-synuclein that was synaptic versus nonsynaptic, we also measured the density of fluorescence at synapses and axoplasm within injected axons. Confocal images were median filtered and then bleed through corrected (bleed through - WT: 4.8–21.4%, pS129: 1.9–31.7%). Injected axons were identified by a freehand mask on the α-synuclein channel and synapses were identified in the SV2 channel using a clustering-based image segmentation method. This mask was then applied to the injected axon mask from the α-synuclein channel to distinguish between synapses and axoplasm. Intensity counts in the α-synuclein channel were subsequently summed for those overlapping with SV2 (synapses) or without SV2 (nonsynaptic/axoplasm), and divided by the total area for each to obtain the fluorescence density. This analysis was written in MATLAB (The MathWorks, R2022b; Natwick, MA).

### Electron microscopy

For EM experiments, fixed spinal cords were processed, embedded in EMbed-812, sectioned at ∼70 nm, and placed on copper Formvar slot grids (EM Sciences; Hatfield, PA), as previously described (Busch *et al.*, 2014; Medeiros *et al.*, 2017; Walsh *et al.*, 2018; Banks *et al.*, 2020). Sections were counterstained with 2% uranyl acetate and 0.4% lead citrate. Synapses within injected axons were imaged at 37,000X magnification on a JEOL JEM - 200CX transmission electron microscope (Peabody, MA) using a Hamamatsu C8484-05G Digital CCD side mount camera (Hamamatsu Photonics; Shizuoka, Japan). Synapse images were analyzed from three regions: control (>400 μm from injection site); low concentration (150–400 μm from injection site); and high concentration (<140 μm from injection site). Based on the diffusion of the coinjected fluorescent dye, we estimate that the final axonal concentration of pS129 α-synuclein in the high and low concentration regions were ∼10–20 μM and ∼5–8 μM, respectively. Control synapses were obtained from distances beyond where the pS129 α-synuclein had diffused. Three-dimensional reconstructions were generated from five serial micrographs using 3D Reconstruct software version 1.1.0.0 (Fiala, 2005).

Morphometric analyses were performed on *n* = 22–67 synapses (*n* = 2–4 axons) per experimental condition by researchers who were blinded to the experimental conditions. For these analyses, all synaptic membranes were quantified in FIJI from a single section obtained at or near the center of the AZ, as previously described (Busch *et al.*, 2014; Banks *et al.*, 2020). These measurements included the number and size of SVs, PM evaginations, Cist, and clathrin-coated pits and vesicles (CCP/Vs). CCP/Vs were further staged as follows: (stage 1: initiation of CCP formation; stage 2: maturation of CCP; stage 3: fission; stage 4: free CCVs/uncoating). PM evaginations were measured as the curved line distances between the edges of the AZ (right and left) and a point at 1 μm straight line distance along the inner axolemma, and then averaged. Vesicles >100 nm in diameter were counted as “Cist”, which are presumptive endosomes. From these measurements, a total membrane analysis was performed (Busch *et al.*, 2014; Banks *et al.*, 2020). The SV distribution and nearest neighbor analyses were performed using codes written in MATLAB (The MathWorks, R2022b; Natwick, MA), which have been deposited in GitHub: https://github.com/jaqulinwallace/MBL-Morgan-Lab-Analysis. In short, for the SV distribution analysis, a curved line distance along the AZ was discretized and used to measure the shortest distance between the center position of each SV and the AZ. Nearest neighbor distances were calculated using the center positions of each SV. Elliptical fits were found using the eigenvalues and eigenvectors of the covariance matrix from the point cloud which defines the elliptical axes and tilt angle through principal component analysis. All statistical analyses were performed using GraphPad Prism 9.5. Graphing was performed using either MATLAB or Prism 9.5.

### FM dye labeling and live imaging

FM 1-43 dye uptake at lamprey giant RS synapses was as previously described (Kay *et al.*, 1999; Photowala *et al.*, 2006; Bleckert *et al.*, 2012). Briefly, axons were first microinjected with Alexa dye (green), and action potentials were evoked with 3 ms depolarizing current pulses through the recording microelectrode. Axons were stimulated (2000 Stimuli, 5 Hz) during superfusion of FM 1-43 (5 µM) and CNQX (5 µM) and AP5 (100 µM) to block postsynaptic responses. After stimulation, FM dye was removed, and excess fluorescence was quenched with Advasep-7 (Cydex Pharmaceuticals; Lexena, KS) (Kay *et al.*, 1999; Bleckert *et al.*, 2012). This left FM 1-43 labeled puncta clearly visible. After FM dye labeling, synapses were subsequently left untreated (control) or alternatively treated with either WT or pS129 α-synuclein via axonal microinjection, as described above. Vesicle trafficking was induced with action potentials (4000 stimuli, 20 Hz), and FM labeled axons were imaged using the lattice light sheet microscope. Z-stacks were taken (10 Z-planes, 5-μm deep; 51 × 51 μm), from which the synaptic destaining kinetics, as well as intensity and movement of FM puncta were measured over time. The number of motile FM puncta, defined as those particles that moved in five or more sequential frames, were measured within a 50 µm length of axon over a 200 s time period during the 20 Hz stimulation from *n* = 22 and 33 synapses (*n* = 3 and 4 axons) per condition.

### Statistics

For all experiments, the experimental sample sizes (n’s) were determined using a biostatistics program: (www.quantitativeskills.com/sisa/calculations/samsize.htm). Except where noted above, all means and standard errors were calculated, statistical analyses performed, and graphing conducted using GraphPad Prism 9. (Figure 2, B and D): For protein-lipid overlay data, no outliers were identified using the ROUT method, and all data passed the D’Agostino and Pearson normality test (α = 0.05; *p* > 0.05); variances were not compared between these datasets because they were generated with two different antibodies with slightly different sensitivities for WT or pS129. (Figure 3, B and D): Membrane binding data were analyzed using an unpaired Student’s *t* test. No outliers were identified by the ROUT method, and all data passed the D’Agostino and Pearson and Shapiro-Wilk normality tests (α = 0.05; *p* > 0.05). Variances were similar between WT and pS129 groups (F test; *p* > 0.05;. Figures 6, D–I; 7, G–O; 8, E–K; Supplemental Figure S4): For all EM analyses, original images were randomized and coded by one researcher and then analyzed by another who was blinded to the experimental conditions. EM data were analyzed using a one-way ANOVA, followed by a Tukey’s post hoc test. No outliers were identified by the ROUT method. Variances between experimental groups were similar for the SVs, Cist, CCP/vs., total membrane, and nearest-neighbor distances (Brown-Forsythe test; *p* > 0.05), but not for PM (Brown-Forsythe test; *p* < 0.05). In most cases the EM data passed the D’Agostino and Pearson normality test (α = 0.05; *p* > 0.05), except for the CCP/V data, due to sparse numbers of these structures. For Figure 8, I–K, an additional bootstrap method was conducted for the elliptical plotting with a randomized set of *n* = 2000 SV distances, which showed that the results for the elliptical plotting held. (Figure 9): For the FM1-43 experiments, decay rates of fluorescence during 20 Hz stimulation were measured by fitting single exponential curves to background subtracted data and compared using a one-way ANOVA. Changes in variance between control, WT-, and pS129-treated synapses were compared using an F-test for variance as reported in Results. Moving puncta were analyzed using *t* tests with Bonferroni correction.

## Supplementary Material



## Abbreviations used

DLB: dementia with Lewy bodies
PD: Parkinson’s disease
PLK2: Polo-like kinase 2
pS129: phosphoserine 129 α-synuclein
RS: reticulospinal
SV: synaptic vesicle
