# Supplementary material for: Excess phosphoserine-129 α-synuclein induces synaptic vesicle trafficking and declustering defects at a vertebrate synapse
Source: Mol Biol Cell. 2023 Nov 22;35(1):ar10. doi: 10.1091/mbc.E23-07-0269 (PMC10881165; doi:10.1091/mbc.E23-07-0269)
Supplement: Supplementary file 3 [file mbc-35-ar10-s001.pdf]

Supplemental Materials

Molecular Biology of the Cell

Wallace *et al*.

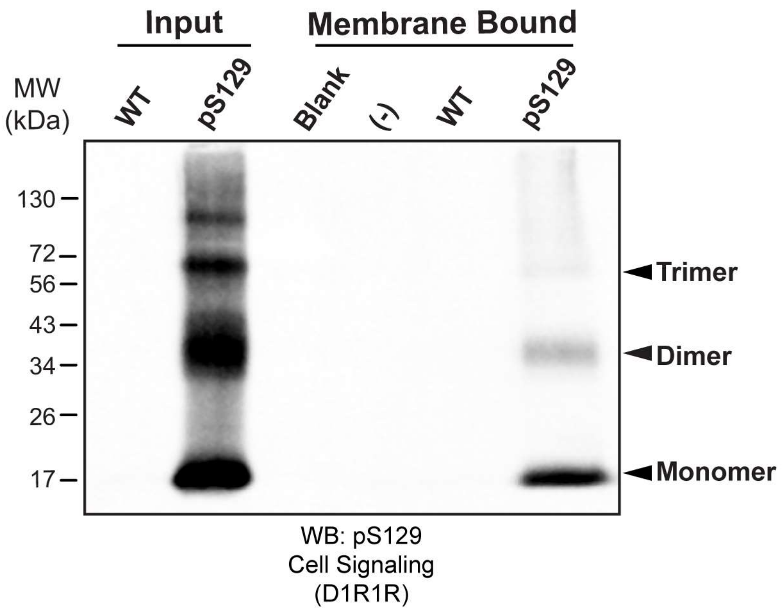

Supplemental Figure 1. WT α-synuclein is not phosphorylated at serine 129 upon membrane binding. Samples from Figure 3 were Western blotted with a pS129 α-synuclein-specific antibody (Cell Signaling; D1R1R; 1:1000). No signal for pS129 was detected in the WT α-synuclein lane, indicating that WT α-synuclein did not become phosphorylated at S129 upon membrane binding. (-) indicates membranes that were incubated with cytosol only and not supplemented with α-synuclein. Image shown is representative of n=2 independent experiments.

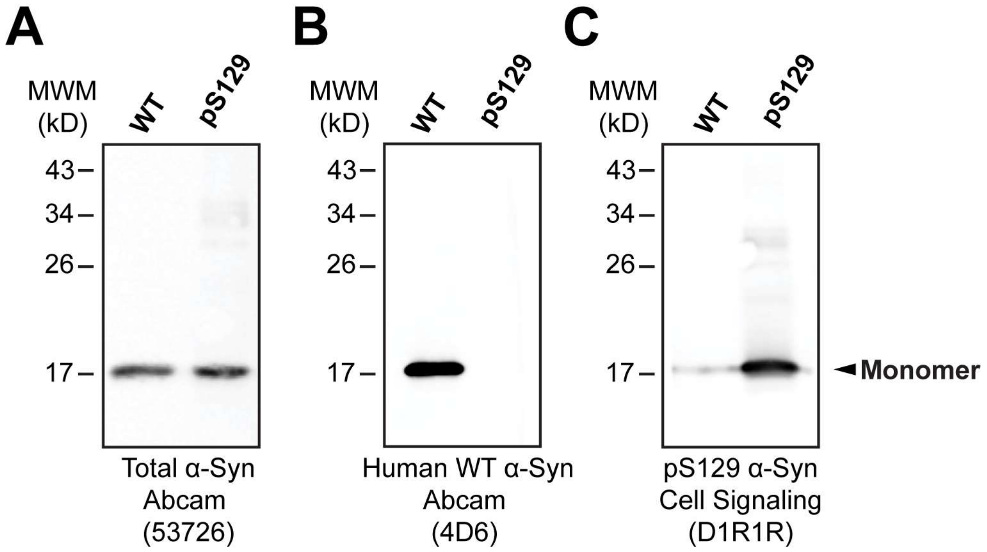

Supplemental Figure 2. Characterization of WT and pS129 α-synuclein used for the *in vitro* SV clustering assays. (A) Western blot of total α-synuclein showing equal loading of recombinant human WT and pS129 (250 ng). Protein sources and purification are as described in Methods. (B-C) Western blotting of the same proteins using WT- or pS129-specific α-synuclein antibodies confirming their identity. All antibodies were used at 1:1000, and sources are as indicated in A-C.

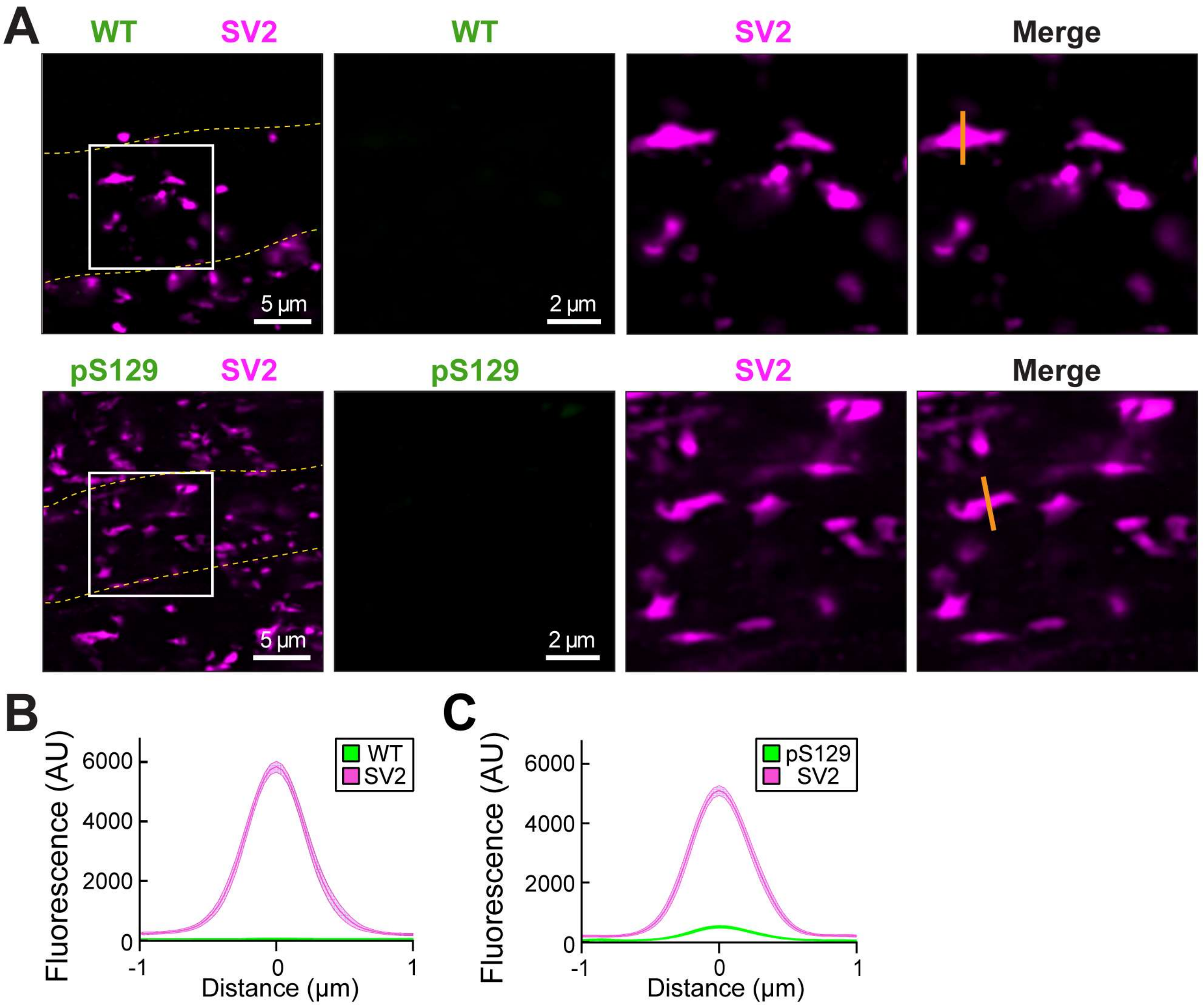

Supplemental Figure 3. Whole mount immunofluorescence on lamprey spinal cords using human α-synuclein antibodies show no reactivity for endogenous synucleins. (A)
Uninjected giant RS axons were immunostained with human α-synuclein-specific antibodies that recognize WT (top: MJFR1 antibody; green) or pS129 (bottom; D1R1R antibody; green), as well as SV2 (magenta). High resolution confocal images were obtained using a Zeiss LSM980 Axio Examiner with Airyscan2 (63X, 1.4 NA objective). Dotted lines indicate border of a giant axon. White box in leftmost panel indicates the ROIs for the subsequent images. Solid line in the rightmost merged image indicates the synaptic position from which the intensity analysis was performed. (B-C) Histogram analysis showed a lack of signal from the human α-synuclein-specific antibodies at the SV clusters within uninjected, control lamprey axons (n=29-30 synapses, 7-10 axons, 2 animals). This confirms that these antibodies are specific for the injected human α-synucleins (see Figure 5).

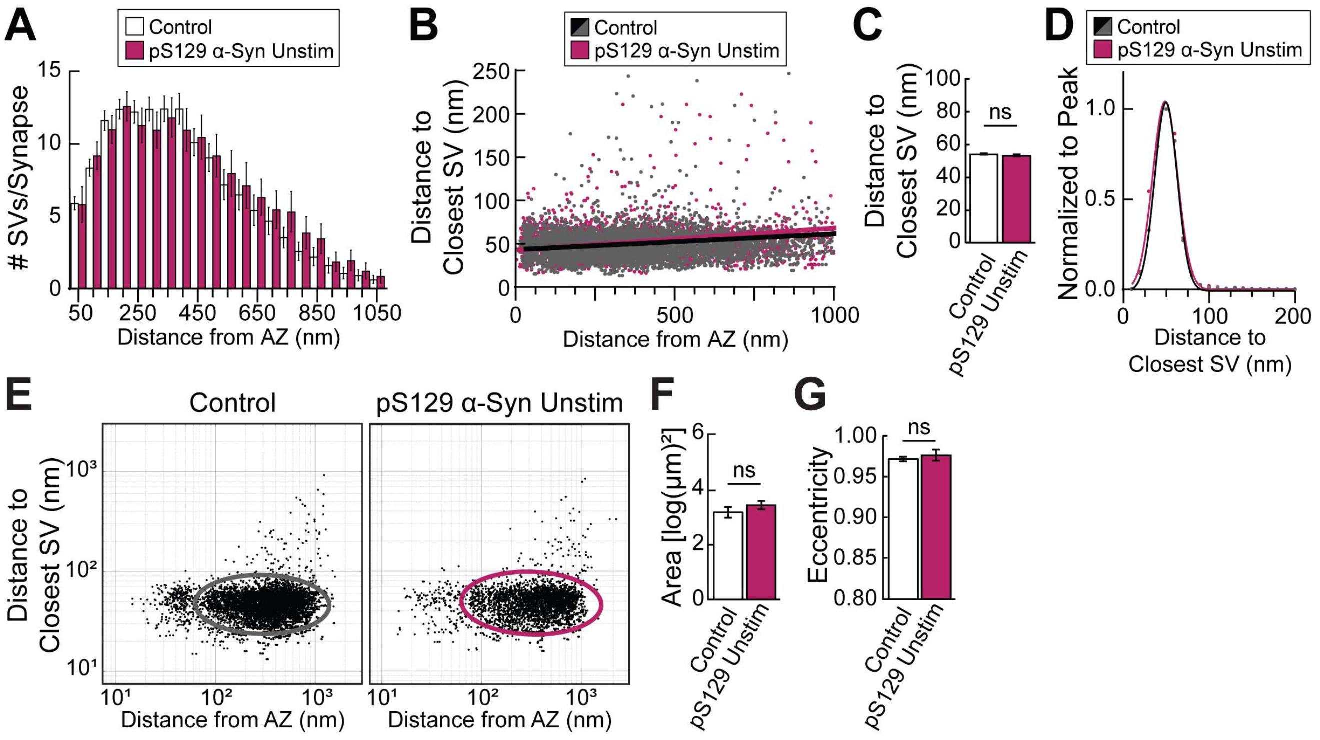

Supplemental Figure 4. Excess pS129 α-synuclein (10-20 μM) does not cause SV declustering at unstimulated lamprey synapses. (A) SV distribution analysis. There was no significant difference observed in the number of SVs found at 50 nm intervals from the AZ between control (white) and pS129 (magenta). (B) Nearest neighbor distances between individual SVs were unchanged with pS129 α-synuclein. [Control: slope=0.01824 ± 0.001671, R^2=0.0212, n=5,504 SVs, 39 synapses, 2 axons; pS129 (unstim): slope=0.02549 ± 0.002402, R^2=0.03301, n=3,302 SVs, n=22 synapses, 2 axons; linear regression; ANOVA p=0.0002]. (C) There was no significant difference in the mean distance between SV at low or high concentrations of pS129 α-synuclein. (Control: x=53.58 ± 0.44, n=6,578 SVs, n=39 synapses, 2 axons; pS129 (Unstim): x=52.76 ± 0.61, n=3,302 SVs n=22 synapses, 2 axons; ANOVA p=0.5085) (D) Nearest neighbor SV distributions were best fit by a single Gaussian curve and were also unchanged with pS129 [Control: R^2=0.9969; pS129 (Unstim): R^2=0.9891]. (E) After converting data in panel B into a log-log plot and fitting an ellipse, the density plot for control versus pS129 are not significantly different. (F-G) Eccentricity and area of the fitted ellipses do not change significantly with increased pS129 concentrations. [Area - Control: 3.17 ± 0.19 log(μm)^2, n=39 synapses, 2 axons; pS129 (Unstim): 3.44 ± 0.14 log(μm)^2, n=22 synapses, 2 axons; ANOVA, p=0.7999; Eccentricity - Control: 0.878 ± 0.017, n=39 synapses, 2 axons; pS129 (Unstim): 0.896 ± 0.034, n=22 synapses, 2 axons; ANOVA, p=0.8512]. n.s. indicates "not significant" by one-way ANOVA.
